# Ripple effects of research capacity strengthening: a study of the effects of a project to support test facilities in three African countries towards Good Laboratory Practice certification

**DOI:** 10.12688/gatesopenres.13190.1

**Published:** 2020-11-27

**Authors:** Sara Begg, Alexandra Wright, Graham Small, Diabate Abdoulaye, William Kisinza, Benjamin Koudou, Sarah Moore, Franklin Mosha, Constant Edi, Matthew Kirby, Patrick Kija, Robert Malima, Jason Moore, Imelda Bates

**Affiliations:** 1 Liverpool School of Tropical Medicine, Liverpool, L3 5QA, UK; 2 London School of Hygiene & Tropical Medicine, London, WC1E 7HT, UK; 3 Innovative Vector Control Consortium, Liverpool, L3 5QA, UK; 4 Institut de Recherche en Sciences de la Santé, Bobo-Dioulasso, Burkina Faso; 5 National Institute of Medical Research, Amani Centre, Muheza, Tanzania; 6 Centre Suisse de Recherches Scientifques en Côte D’Ivoire, Abidjan, Cote d'Ivoire; 7 Ifakara Health Institute, Ifakara, Tanzania; 8 KCMUCo-PAMVERC, Moshi, Tanzania

**Keywords:** Laboratory, research capacity strengthening, good laboratory practice, insecticide, test facility, quality management system, quality management systems, capacity strengthening

## Abstract

**Background: **Strengthening capacity for public health research is essential to the generation of high-quality, reliable scientific data. This study focuses on a research capacity strengthening project supporting seven test facilities in Africa conducting studies on mosquito vector control products towards Good Laboratory Practice (GLP) certification. It captures the primary effects of the project on each facility’s research capacity, the secondary effects at the individual and institutional level, and the ripple effects that extend beyond the research system. The relationships between effects at different levels are identified and compared to an existing framework for the evaluation of research capacity strengthening initiatives.

**Methods:** To capture the views of individuals engaged in the project at all levels within each facility, a maximum-variation purposive sampling strategy was used. This allowed triangulation between different data sources. Semi-structured interviews were conducted with individuals in three facilities and a combination of email and remote video-call interviews were conducted with individuals at two further facilities.

**Results:** We found that, despite a focus of the GLP certification project at the institutional level, the project had effects also at individual (including enhanced motivation, furtherment of careers) and national/international levels (including development of regional expertise). In addition, we detected ripple effects of the project which extended beyond the research system.

**Conclusion:** This study shows that research capacity strengthening interventions that are focussed on institutional level goals require actions also at individual and national/international levels. The effects of engagement at all three levels can be amplified by collaborative actions at the national/international level. These findings show that research capacity strengthening projects must develop plans that address and evaluate impact at all three levels. Capturing the ripple effects of investment in research capacity strengthening should also be planned for from the beginning of projects to support further engagement of all stakeholders.

## Introduction

Building research capacity in public health and related fields is essential to the generation of robust, innovative and locally relevant scientific data. When research staff are highly skilled and research infrastructure at institutions is strong, the evidence generated by these institutions can inform national policies, support progress towards population health goals and contribute to socioeconomic development ^1– 4^. Research capacity strengthening is increasingly an area of focus for international development and global health partners and funding bodies ^5, 6^. With increasing investment of funds to support research capacity strengthening, there comes an increased need to evaluate the impact of this investment on data quality ^7^. Test facilities are a key component of national research capacity. Attention is commonly focused on clinical diagnostic and research facilities, their role in diagnosis and support in disease and epidemiological surveys ^8^. However, non-clinical and basic science facilities also have key roles to play in global health research ^9^. This can include supporting entomological mapping surveys such as insecticide resistance mapping, generating scientific evidence that can inform the discovery of novel compounds for therapies, development of new products that may have uses in public health, including the control of vectors of diseases, and assessing the safety of these compounds and products before they are used.

This study focuses on a research capacity strengthening project supporting seven test facilities in Africa towards full compliance with Organisation for Economic Co-operation and Development (OECD) principles of Good Laboratory Practice (GLP) ^10^. These test facilities are all engaged in the evaluation of mosquito vector control products, including long-lasting insecticidal nets and indoor residual spraying formulations ^11^. Each test facility consists of an insecticide testing facility (ITF), a molecular biology laboratory, experimental hut sites, an insectary, and animal houses. Data generated by these test facilities inform decision making at a national and international level, as these test facilities have historically conducted laboratory and field efficacy trials on vector control products for evaluation by the WHO Pesticide Evaluation Scheme (WHOPES) ^12^ which supported national programmes and other stakeholders in the selection and safe and judicious use of public health pesticides. With ever-mounting challenges related to increasing insecticide resistance and changes in vector profile and distribution due to climate change, there is a pressing need for innovative vector control products, tools and approaches. To support this, WHO has now transitioned the function for evaluating these products to the WHO Pre-Qualification Vector Control Team (WHO PQ-VCT), to align the quality assurance of vector control products with existing prequalification processes within WHO ^13^. Test facilities will now generate data on behalf of companies for the evaluation and prequalified listing of vector control products by WHO PQ-VCT, which guides UN agencies, other international organizations and country-level procurement bodies on the procurement of products for malaria management and eradication ^14^. Whilst test facilities are moving towards GLP certification, WHO PQ-VCT can inspect data-generating facilities to ensure quality data. However, once sufficient test facilities have been granted GLP certification, WHO PQ-VCT will require companies ‘to develop a product dossier which includes data and information to support the safety, efficacy, and quality requirements appropriate to the product type and generated according to Good Laboratory Practices (GLP) and appropriate Quality Management System (QMS)’ ^15^. The conducted of studies compliant with GLP principles will ensure that data generated for product registration purposes are reliable, reproducible and auditable and will be recognised by scientists and regulatory authorities worldwide. Each test facility was supported towards GLP certification by the Innovative Vector Control Consortium (IVCC), with funding from the Bill & Melinda Gates Foundation being used to support the development and implementation of quality management systems, infrastructure improvements, facility inspections to identify and address nonconformances with GLP principles and staff training activities.

Research capacity strengthening has been defined as ‘a process by which individuals, organisations, and society develop the ability to perform [research] functions effectively, efficiently and in a sustainable manner to define objectives and priorities, build sustainable institutions and bring solutions to key national problems’ ^16^. This definition highlights that research capacity strengthening happens at three levels: the individual level, the organisational or institutional level, and the societal or national/international level. In capacity strengthening, initiatives are often focused at one of these three levels ^8, 17^, with programme goals and evaluation of programme success aligning directly with these levels. In this study, the described goal was at the institutional level – developing a QMS compliant with the principles of OECD GLP and being granted GLP certification. Despite an institutional-level goal, the interventions required to implement this system acted at individual, institutional, and national/international levels.

The purpose of this study was to capture both the primary effects of the GLP certification project on each institution’s research capacity, the secondary effects at the individual and institutional level, and any ripple effects beyond the research system. The relationships between effects at different levels are identified. These effects are compared to an existing framework for the evaluation of research capacity strengthening initiatives, to identify new areas for future laboratory capacity strengthening programmes to consider when developing and evaluating their interventions. In addition, we saw ripple effects of the project beyond research capacity strengthening for both individuals within each facility and into the community surrounding them.

## Methods

### Ethical statement

Ethical approval to conduct this research study was obtained from the Liverpool School of Tropical Medicine Research Ethics Committee (approval number 18-041), the National Institute for Medical Research Tanzania (ref NIMR/HQ/R.8c/Vol./I/554), and the Centre Suisse de Recherches Scientifiques en Côte d'Ivoire Institute Review Board (ref 19-549). Institutions taking part remotely (i.e., interviews with members of research staff via Skype/email) provided an institutional approval document in lieu of in-country REC approval, as per point 3c of the LSTM’s Approval Processes for Network and Capacity Strengthening Studies.

Participants were informed about the research using participant information sheets ^18^. Written consent was obtained from each participant prior to undertaking an interview.

### Setting

Seven insecticide test facilities engaged in the testing of novel vector control products for the purpose of supporting malaria control programmes have received investment and support from IVCC to achieve GLP certification. Of these seven facilities, five have been included in this study, encompassing test facilities in Tanzania, Côte D’Ivoire and Burkina Faso. These five test facilities encompass a diverse array of contexts. PAMVERC-KCMUCo, Tanzania, provides crucial information on how GLP certification can be achieved, being the first insecticide testing facility in Africa to do so. Comparison between East and West African contexts was facilitated through inclusion of Centre Suisse de Recherches Scientifques en Côte D’Ivoire (CSRS) and Institut de Recherche en Sciences de la Santé (IRSS), Burkina Faso. Comparison between government and non-government test facilities was facilitated through inclusion of National institute For Medical Research (NIMR), Amani Centre, Tanzania and Ifakara Health Institute (IHI), Tanzania. These contrasting test facilities enhanced our ability to identify both direct and indirect effects of investments in developing a QMS. Generalisability of findings was assessed through using these facilities to compare effects of investment in QMS in a diverse range of contexts, including different national policy contexts and government/non-government supported test facilities.

### Sampling

To capture the views of individuals who had exposure to the GLP certification process at all levels of these test facilities, a maximum-variation purposive sampling strategy was used ^19^. Sampling included those who hold key roles within a test facility, as determined by a case-study conducted on the first test facility to achieve GLP certification, KCMUCo-PAMVERC ^20^, as well as multiple representatives at each organisational level of the facility. This allowed triangulation between different data sources to determine the trustworthiness of findings. Test facility organograms were used to identify relevant participants, with guidance from stakeholders at IVCC and GLP project managers.

### Data collection and analysis

Semi-structured interviews were conducted with individual staff members involved in the GLP process in three test facilities; KCMUCo-PAMVERC, NIMR Amani Centre, and CSRS. The interview topic guide ^18^ was developed based on previous studies of laboratory capacity strengthening ^8^, with additional questions derived from findings from a case study of the GLP certification process at PAMVERC-KCMUCo ^20^. One overarching question was specifically related to perceived effects of the project. However, due to the semi-structured nature of the interview, interview participants reflected on the effect of the project throughout the interview. Specific questions asked from the topic guide were matched to the roles and responsibilities of the interviewee. Interviews were audio-recorded and transcribed in full. All interviews were conducted in person, in a private room or office, by two researchers, one of whom had a technical understanding of GLP requirements in insecticide testing facilities and the other having systems evaluation experience. Whilst the lead researcher spoke basic French and Swahili, for interview participants who preferred to undertake the interview in a language other than English, a trusted colleague or research student sat in on the interview to aid with translation.

A combination of email and remote video-call interviews were conducted with individual staff members involved in the GLP process at two other test facilities, IRSS and IHI. This was necessitated by restrictions on travel and reduced working hours following the COVID-19 pandemic, which resulted in significant disruption from March 2019. The overarching questions asked in these interviews were retained from the semi-structured interview guide used for in-person interviews. Follow-up questions, where relevant, were conducted via video-call or email.

A framework analysis ^21^ was used to identify themes emerging from the interview transcripts following the five-step process of familiarization, identification of thematic framework, indexing, charting and mapping/interpretation. The framework identified was the Research Capacity Strengthening evaluation framework developed by Khisa *et al.*, from African Population and Health Research Center, Nairobi, Kenya and Centre for Capacity Research, Liverpool School of Tropical Medicine, UK ^22^. This framework delineates the identified and envisioned effect of research capacity strengthening initiatives at the individual, institutional, and national/international level, developed from a review of the research capacity strengthening literature and refined in consultation with research capacity strengthening funders, implementers, managers and evaluators ( Table 1). Following familiarisation with the interview data, further themes were identified and incorporated into the framework, while retaining the individual, institution, and societal level structure. All interview transcripts were indexed using NVivo software version 11 (QSR International).

**Table 1.** Framework for evaluating Research Capacity Strengthening from Khisa *et al.*, 2019 ^22^.

Institutional level	Individual level	National/international level
Career pathways for the research team	Provision and quality of training for the research team	National: research councils/research productivity
Sustainable provision of appropriate, high quality training	Recognition of research leadership/esteem	International: networks/ collaborations
Nationally/internationally competitive research and grants	Career trajectory	Research effect and user engagement
Research environment – finance, library, IT, labs etc		

## Results

A total of 65 members of staff from five test facilities participated in this study. 66 were approached to take part, with one declining to take part. Of these staff, 16 were laboratory/insectary technicians or attendants, 17 were from non-scientific administration/information technology positions, 22 were from scientific middle-management positions, and 11 were from scientific senior management positions. 49 were male and 16 were female. Anonymised identifiers have been used for quotes from transcripts, highlighting the role of the interview participant but not the test facility they are connected to. These are presented in Table 2 and referenced by section in the text.

**Table 2.** Target level for RCS.

Individual Level						
Provision and quality of training for the research team (IND1)	Our technician also flow now especially, even before it was difficult to verify and to check if they follow or not, but now we are checking, yes. Our technicians were also trained. They received training on how to apply the SOP and so on. *Study* *Director*	Translator: She said that the training has changed her. She knows when she comes to work, she knows what she's going to start with also it's doing cleanliness. She has to document everything and the like. Yes. *Technician*	We had some training from [IVCC member of staff 1] on the development of SOPs. We had some training from [IVCC member of staff 2]. We had also training with other people-- the GLP managers, active people. I also had training on GLP which was conducted in Moshi. That was the first one. It was big because it combined different sites. We were with the people from West Africa. That training was perfect. It was very nice. It exposed us to this process, why do we need to do it, why should we change our attitudes towards what we are doing, what's the value of it. It was very nice. *Laboratory* *Supervisor*	We have other people from IVCC that we've attended a meeting on this workshop on how to sustain the GLP facilities. We got a lot of training and actually I attended that was present and that was very, very productive. We wish that there should be continuously because we acquired a lot of knowledge of how to sustain our negotiation skills with the clients and everything like that, so on how to plan and so we got a lot. *Test Facility Manager*	Even training have increased. Before GLP we didn't have even health and safety things, but now we see people are being trained to go do the health and safety issues. Such things were not there before. *Laboratory * *Supervisor*	We initially conducted external training for all staff on general GLP principles from [IVCC member of staff 2] which was useful as it put all levels of staff on a strong foundation. *Test* *Facility Manager*
Recognition of research leadership/ esteem (IND2)	Translator: For him personally, GLP, the lesson he got about GLP it make him to honour his work. If he’s not around, anybody can do his work or he can know what work he can do. Nobody will match their own work. You will know your work, your role, what to play, what to do. *Administrator*	The main challenge that we have now is implementation of these new SOPs to make sure that everybody adhere to the SOP. We are happy. Things are moving. We see that we are doing science now. *Laboratory* *Supervisor*	I think the team as well would be happy to see the products which have been evaluated here and found to be effective as seen in the market. This is an indirect benefit to see the products. I’m saying this because I’ve been involved in evaluating a number of these products. When I go out there even in other missions and I found those products in the market, it's a great feeling and I can tell the story. Indirectly, that is the main benefit. *GLP* *Project Coordinator*	I think the opportunity for the staff to grow and become better science wise has improved. I don't know but going to GLP should also be on everyone's CV hopefully. *Study Director*	Benefits are there because, for example, personally, I feel good to be working in a GLP accredited institution. Whenever I go, for example, in the old institution that I used to work, they see me different just because I'm here and working. *Laboratory* *Supervisor*	Yes, because they are now profession. Profession everything-- when you do something and you see this one, "Yes, I've done it." It's one of the professionalism. *Technician*
Career trajectory (IND3)	Also, while doing that, it will form the well-being of each person, of each staff, because everybody wil involve and will gain at all level, not only one person or administration that will gain more than any part of the services. *Administrator*	I must admit that in the government system, we don't train these people that much. Here, the system was good for scientists and technicians but not for them. They were called supportive staff. With GLP, at least they're now considered. They get training on what to do, which is very good for their career as well. *Research Scientist*	I think is through hard work because I was employed here just as a technician and then I was promoted to that position. I think it's how I was working that- and my educational qualification, the combination of two maybe brought out that position. *Laboratory supervisor*	Yes, for instance, it's good in your resume if you're working in a place which has accreditation, it's a big plus. It means you're fit to work there. *Technician*	We did recruit specifically for a GLP coordinator externally which was not the correct fit and this position was later filled internally. *Test* *Facility Manager*	
Structured work practices (IND4)	Translator: For him, GLP helped him to update more things, to organize more things. It's helped him to do good practice and help people to find out the gap and create it. *Technician*	Translator: They taught a lot. Yes, for him, for his concern, they have taught a lot about the GLP to them. Now it's like it's incorporate into their body, you have to practice the GLP he says. GLP will help them to order, organize things, to understand more the procedure of doing things, and to practice ahead of it in case someone is not around, maybe on holiday or got sick, they can follow and do the work, because there is a manual, there is a procedure, everybody can follow and you can do the work easy. *Administrator*	I see there is a very positive impact because as you see, GLP is all about people. I wouldn’t say SOPs. Is insisting about the SOPs, what people are supposed to do. Therefore, I see it has a positive impact to me because people will be more, should I say proactive? *Administrator*	Because of how GLP wants you to move things, help you to be creative. To be creative, so that you can do what you are supposed to do. That has helped me a lot. To manage, to manage the people you're working with. *Laboratory* *Supervisor*	Translator: She says it helps because formerly, they use it let's say for cleanliness, they use it to do routine cleanliness without knowing that maybe the coming days they are going through the same or what is next. Therefore it has helped, it has changed the household because it has become more systematic and it is specified. *Technician*	I think I actually learned a better way of how to maintain or how to keep track of what I'm doing. This has actually been a good way from you actually know like everything where everything is and if I want to remember something, I don't have to actually guess about it. I have a log of everything that I have done. *Administrator*
Transfer of organisation skills to home (IND5)	When we talk about policy, it is the best thing that give us some kind of governing life because apart from being here working with GLP, it help also us to know that in life you have to follow some guideline, and you have to do things followed by some rules. Personally, it is more instructive. Yes. *GLP Manager*	GLP also is teaching us how to be punctual. Not punctual only in the working place, but in your family. GLP is helping us to save, in saving, in budgeting. That is something which somebody can't see, so is to me is indirect benefit. *Laboratory Supervisor*	I find it very useful because [GLP project manager], actually, helped us to change their mind; because to move on with this facility, people need to change the mind. Previously, we were running our business as usual but now we have to change, to be serious, to work hard, and follow the procedures, the SOP for running, for planning different activities. I find it very useful, very useful and it will be applicable not only for GLP but also for my daily activities. *Data Manager*			
Institutional level						
Career pathways for the research team (INS1)	There is what we call performance appraisal. Normally we appraise people quarterly. Every three months. When it comes to performance appraisal, I do find things they are moving because people they have to fill the forms and the like. When you see the comments from the head of department, you find heads of department are doing their part. Even the staff are doing their part. I find it has made my work easy. *Administrator*	More studies doing here. It means people will be busy, they will work because I used to tell people when you work you have to feel proud of because if you don't work, there is a problem. Therefore, I used to tell them if you get updated we are expecting to get much work, to get more studies coming here and if more studies come here we'll get something. *Administrator*	People, I think, generally wants to be trained more. Maybe that desire always existed but there wasn't a channel for people to voice that and now there is. We have appraisals, we have the training committee. *Study* *Director*	For the study that goes across all of the test facility. I think it's a lot clearer for the staff on who does what and who has responsibility for what. I think we have been able to delegate a lot more responsibility because there was a system in place. There's clearer lines of communication. I also think a lot more staff because the staff take on more responsibility, we can do more things. *Study Director*	GLP project has brought new organization with job description for each team member thus facilitating the conduct of activities and the management of the different related issues. *Laboratory* *Supervisor*	
Sustainable provision of appropriate, high quality training (INS2)	Internally, there have been trainings on GLP several times. Those trainings concerned general aspects of GLP and specific aspects such as writing SOPs and their use. Those internal trainings were done at our institution by the quality manager and the supervisors. *Laboratory* *Supervisor*	The seminars. We’re trying to impact them with the new knowledge on how to do things in a standard way. Of course, what we normally do is we have a weekly arrangement that we gather for two hours, we do the presentations, we discuss. *GLP Project* *Coordinator*	because GLP involved everybody in the institution from the director to the super, so we had this phase we’re training for everybody to understand what it is. Then we have this special training for special groups. Like for the lab guys and we have some for administration. *Laboratory* *Supervisor*	Also, it advertise the college in one way or another way as well. We have been training some the colleges on master's level, they've been attached here for their master's as well. *GLP* *Project Coordinator*	I think staff are benefiting getting the training. As of now, we're having a training committee to suggest or to discuss the training request for staffs. I'll say up to now our request which has been submitted to the training committee has been approved, so it's benefit to the staff. *Administrator*	Seriously, the technicality has given the mandate to take all the inquiries and recommend. Even in small budget is set for them as well to facilitate paying for the courses, and set of things. That care structure make perfect kind of obliged to make sure set amount of money to train this people which they perfect. Before then, I think it's up to you to apply at that time manage available. If not, try next time. Now, I think it's easy and it's there. This is relevant to your department, why not take it? I think that's a major benefit. *Quality Assurance* *Manager*
Nationally/ internationally competitive research and grants (INS3)	Yes, rigorous, so this may help us to produce good data, because our technician and the other team will follow this guideline very strictly For me this will put added value into our research capacity. *Administrator*	That is one of the success that we had. Also, the other issue is that we managed to attract some- -To get some interaction with clients and looking for our technical support and the evaluation of their products. For instance, for the first phase one evaluation of products-- Since the inception of the workshop in Liverpool, we had about three-phase two studies-- Phase one. *Test Facility Manager*	Yes, for example, right after coming back from the training, from Moshi, there was one project that was going on. I started doing those small procedures in how to manage data and how to collect the data in a proper mechanism. Also, doing, for example,- in that training also they insisted how to use the double-entry, which you are not doing, but now we are doing four projects doing the double entry. *Data Manager*	Well, the general quality of research even for the non-GLP studies is very similar to the GLP. We run them and file them pretty much. I think the staff are much more keen on the system, the processes, they're much more interested in the work. *Study Director*	In fact, before implementing those documents in the insectary, there were frequently issues and whenever they were occurring we (technicians and I) had difficulties to identify the origin of the problem. But, since we have used those documents, issues occur very rarely and even if issue occurs the interesting thing is that we identify easily its origin by checking if there is not something in the SOPs, guidelines or forms that has not been respected. *Laboratory* *Supervisor*	we can give data that is trustworthy since it is collected in a defined standards and by using well maintained / validated equipment, and most of all the output of good quality data from research is for the benefit of the whole community i.e. when we say a certain product is efficacious then it’s really so this then mean protection of the whole public and vice versa etc. *Quality Assurance* *Manager*
Research environment – infrastructure (INS4a)	I think that before you work in different projects but there is somethings who was not sometime adapted to the entomology. I think now there is some infrastructures like when you go to the insectarium and to the lab you see there is new materials. There is notifications of the meetings and also there is archive office. There is also in [Field Site] there is a new building. We see that there is some evolution. *Laboratory* *Supervisor*	Yes, so we refurbished the archiving room. We refurbish our testing lab, and secretary as well. We build, add more house close to accountancy. That animal house. We also build some room for net washing at phase one. *GLP Project Coordinator*	Yes, very rewarding I would say; very, very rewarding. Not only in [Field Site]; even here, very rewarding. You can see now the condition of working for every one of us-- scientists, the technicians, the assistants-- has been very nice drastically. Even there at the [Field Site], the rooms now, they are very comfortable for the people who are sleeping; very comfortable. Previously before this GLP, we had some instances where bees would invade the hut and stay there. *Laboratory Supervisor*	Well, from me, because I used to be here for the long time and I know what the structure, how it looks. For sure now we have a good structure for GLP. Because everywhere it changed from the secretariat, from the laboratory, from our molecular lab. Five, six years ago you don’t a molecular lab, but now we have. Another thing that is very good for us, some of the tests especially PCR and like that was take our specimen to [National Laboratory] for performing out tests there. From now, from this year we didn't take to [National Laboratory] again. We perform our tests here. *Technician*	It was very big advantage for the college because the grants that enabled us to become GLP compliant also enables us to restructure the buildings, which is not the expense from the college itself, but it's from the grants that came for GLP compliance. It's an advantage for the college to have a facility up to GLP standards and not from their budget, actually, so is advantage. *GLP* *Project Coordinator*	To meet the GLP requirements, a new building was built with the financial and technical support of IVCC. This building has all the necessary facilities and is equipped according to GLP requirements. To increase our capacity in terms of field work (phase II study) we have also built new experimental huts in addition to old ones. *Laboratory* *Supervisor*
Research environment (b) – IT, human resources, procurement (INS4b)	We don't make the SOPs because we have a process manual here. Then all the rules is inside. That is not very clear and not all of us know the process then we don't make it very clear like SOPs. With this process we have now-- we realize that we have to write all the process it is not only for the GLP, it's for all the things you do, it's only for the procurement, it's not for the secretary, it's not for the [French language] because the process is that [French language]. [French language] Outside GLP and manual I write it for myself or for my team, the SOPs for procurement process. Now for us, we're writing with my team to know even if it's my colleague with I was in-charge of these services then when she was away on holidays. Another person can take the process and make the same thing. *Administrator*	In volume paper. We don't needing looking at regularly but for SOPs is more short. You can read it easily. Interviewer: Have the SOPs had an impact on your work? Interviewee: Yes. It impact because I know already what I have to do but how to explain it to the new person who come to meet me, it become more easy. *Administrator*	Yes. I will explain it. Each services has its way of doing their work, but according to SOP, what you think you must do, you're not doing it, so SOP guide them to more of each task. For instance, we had processes in accounts, but they have a lot of papers, documents that they have to validate, if it is to procure some material, some things, it will come to their site, they will just validate. But regarding to SOP, you know what to validate, what you shouldn't validate. So, it's kind of a guiding that help them, each service interacts more smoothly. *Administrator*	I'll actually say some of the computers are not really that expensive but the major part is having the main primary place where you can actually do everything. For us, it made it a bit easier for us to control like most of our research things that we do. We have actually created easier ways like a formal way on which we can actually access things. I think with the findings and everything that we've got it, it should help a lot with putting up research. *Data* *Manager*	Yes, very much because you can easily send information through email instead of looking for everyone. In specific communication has been very much improved. We have telephone system within this building. If I need something from the other, I just call. Even if it's in in the lab. I just called the other department, I tell them I need this, it's there. *Laboratory* *Supervisor,*	
Structured working practices (INS5)	First, we have learned to be accountable. I myself I have learned to value every [national currency] that we get; value for money. The way we used to work before GLP is quite different. GLP money has done more than what we expected it could do, after starting, working with the seriousness and making sure that everything is being delivered, making sure that we get standard material things, things like that. *Laboratory Supervisor*	I believe we have actually had a standardized way of doing things which has really helped us. It has really helped us. We have a standard way of operating everything. Because of how we started the process, it has made us adapt to some of the methods on which you should use to do it. I think it has given us a good way of defining things, how we do, asking things and knowing how to implement new things into the system. *Administrator*	Before that we had someone, let's say the supervision level, maybe temperatures be up to this range. As for now, we are recording it, so it's clear. You can see this is in range or not in range, but before it was not clear that way. You can have history of maybe environmental condition. Maybe we had the problem of mosquito knockdown maybe in the last week-- In the week of February and say, "Okay, is it the same problem?". You can go through records and, "Okay. I think, no. By the time we had this problem, temperatures were maybe out of range. Now it's in range, so it's not the issue of temperature. It's something else." *Technician*	It helped the management and technical team to focus efforts in a more structured way for general working practises and enabled full traceability of test items and experiments. *Test Facility* *Manager*		
Societal (national/ international level)						
National: research councils/research productivity (NAT1)	think we have the support at a high level from the ministry of health and in many meeting when we say that we're going through this process a lot of institutions are happy for us. *Director*	For example, we have a centre for medical entomology and veterinary they really want us to train their students to use our lab and there is the National Institute for Public hygiene they're doing a lot of entomology survey and they want us to process the sample. I told you about the PMI project, so we're using the lab to process the mosquito sample we collect. *Director*	You can see that GLP becomes very interesting. It becomes a centre of excellence for training in this area. These government universities prefer to go to a government institution or centre. *Laboratory Supervisor*	What we think and what we are looking for, what we'll be very happy to see is that the way we do work here in [Test Facility] should be translated to other centres. [Umbrella Institution] has more than 10 centres. We are not the only one who are doing entomology. We have at least other four centres doing entomology. [3 other national facilities], they are all doing entomology. We wish that they should also learn from us and start working using some good SOPs. We wish to share this knowledge to the entire [Umbrella Institution] as an institution and improve it. *Test Facility Manager*	Externally, we received trainings from local collaborative institutions on some specific aspects of GLP such as waste management, biosecurity, use and maintenance of some of our equipment. *Laboratory* *Supervisor*	
International: networks/ collaborations (NAT2)	The first meeting, the [Collaborating Test Facility] meeting was mainly based on to help us to understand the role of the GLP and to also learn from the experience of [Collaborating Test Facility] and how we can do the experience to our facilities. *GLP Project* *Coordinator*	We visited [Collaborating Test Facility] to see how they have- how they have gone, how far have they gone, and what challenges they did. We learned from them, actually. *Laboratory* *Supervisor,*	There are inter-relation of several people that actually they put on this but also learning from other institutions like [Test Facility] as a network with other institution like we have local institution like [Collaborating Test Facility] whom we have already been accredited because we have collaboration with [Vector Control Network], we are working with them, we have the network from West Africa and also there are other people from [UK Institution] actually because our long collaborators. We get a lot of technical support from different people on terms of advice and what because what their actions make sure if the site is well equipped and well GLP- based, that's where we can work properly. *Test Facility Manager,*	Yes, definitely because actually as [Test Facility], we have been supported and we come with GLP centre definitely will have to build capacity within the country and not only within the country even beyond, we're responsible. In [Country], we have several institutions and several researchers so we'll have to build capacity so that will be a seeding place to provide tentacles to other people, make sure that we should be proud of only [Test Facility] but also we should make sure that we disseminate what we have to other people. Make sure that what we are doing should be uniform and whatever is being done in [Country] or somewhere else, wherever our support is needed we are ready to do that. *Test* *Facility Manager*	That's another way and even other institutions, they look us differently. They come and learn from us. We have people coming to learn from us and other will like even to send people to teach them. That's something good. *Laboratory* *Supervisor*	Yes. Actually, because through the GLP process we got connection with many, many companies with many, many organizations. We were able to get support to run the project and get recognition as well out of that. *Director*
Research impact and user engagement (NAT3)	As [Test Facility], I think the main benefit is that, let’s say, is to provide, we normally say that if you don’t have data, you don’t have the right to speak, to provide data which will actually influence the policy change for vector control in the country. That’s the direct. Also, to contribute to the world. To contribute to the world. *GLP Project* *Coordinator,*	The other tissue on the government through the Ministry of Health now they are anxious because the [Umbrella Institution] is the technical arm of the Ministry of Health, so they are looking forward to make sure they have a very competent technical arm that can provide good advice related with vector control and evaluation of new vector controlled tools. *Test* *Facility Manger,*				
Effect of investment in surrounding community (NAT4)	Those people, they really liked the project. The volunteers has been there, and things like that. It was very simple for us to get some people to work there. The same people from the environment were very happy to be involved in part of the work. They said, “It's useful for us. Our kids stay there. They live there.” It was easy to recruit people for working there in the site. Very easy. *Laboratory* *Supervisor*	The village leader was very helpful. The fact that we are also helping to improve the road that they normally pass, that was the moment I liked the most. *Laboratory* *Supervisor*	The renovation activity which has been done here benefitted the entire community of [Region]. The business people who we are working with, the retailer shops where we were getting materials for the construction, the people here who worked here, we all benefited from this. That money went to them, actually, we had to buy materials and to construct. It was a very nice experience for them. We had good relationship with them during the entire process. Even there at the villages, the way we renovated our buildings, the way we are taking care of the road to reach there, the way we live well with those-- because of the projects that keeps the volunteers, they get some incentives, things like that, it has been very nice. They got the benefit out of it. *GLP* *Project Coordinator*			

From the interviews, the research capacity strengthening effect of the programme at the institutional level was consistently identified. These primary effects were particularly evident in the research environment, both physical and administrative, sustainable provision of high-quality training, and the capacity of the test facility to deliver competitive research, i.e. GLP-compliant studies. There were also secondary effects identified at both the individual and national/international level. At the individual level these effects were related to the training delivered as part of the GLP project, but there was also a positive relationship between the strengthened research environment and individual level motivation and job satisfaction. At the national/international level networks between institutions were developed, which also had the effect of further strengthening individual test facilities (institutions) as inter-facility learning was made possible. This meant lessons from test facilities at more advanced stages in the process could be applied to those at earlier stages.

### Institutional level effects

At the institutional level, the GLP quality management system, infrastructural improvements of laboratories and offices, development of clearer and more effective organisational structures, more staff employed, and the transfer of GLP-standard practices to other studies were all identified as research capacity strengthening effects resulting from the GLP project.

The development of a GLP-compliant quality management system and, at some test facilities, the achievement of GLP certification following inspection by the GLP monitoring authority SANAS, is a clear outcome of the work undertaken through the IVCC project. Of the seven test facilities included in the wider project, two have achieved GLP certification to date, and four have submitted their application for GLP certification to SANAS. As a result of GLP certification, these two test facilities were able to deliver national/internationally competitive research, with data meeting international standards. This effect extended also to non-GLP studies conducted at these test facilities, as best practice from GLP studies was applied also to non-GLP studies by both scientists involved in the GLP project and other scientists within the institution, particularly with respect to study documentation and use of Standard Operating Procedures (SOPs). Thus, the overall quality of data generated at these test facilities was enhanced. (Quotes: INS3) Test facilities also identified broader effects on working practices, resulting from the implementation of GLP standards. In particular, increased structure in working practices resulting in a range of benefits including cost savings on reagents, more effective problem solving, and better organisation of work throughout the test facility. (Quotes: INS5)

Career pathways were enhanced by strengthening the processes, policies, and documentation that surrounded organisational structure and human resources. Development of clear organisational structures facilitated communication between individuals in different departments and at different levels within the test facility. This was supported through development and implementation of key SOPs for regular, documented human resource support including appraisals and Curriculum Vitae review. Together, these had an additional effect on individuals’ sense of place and therefore, sense of value within the test facility. In some test facilities, new structures were put in place for requesting training for career development, and staff were adequately empowered to take up these opportunities. Across test facilities, but particularly in those that had achieved GLP certification, there were more job opportunities at the institution, with more studies an investment attracted to the test facility. (Quotes: INS1)

In-house training programmes were developed and delivered across test facilities including general training in GLP awareness, Quality Assurance, training in SOPs, Health and Safety/Fire training, archiving training, leadership training, and computer system validation and usage. Training programmes were often developed by test facility staff following attendance at externally delivered training courses. Implementation of training was overseen by staff in a range of roles, as a result of the additional responsibilities being taken on by staff at all levels. Test facility management noted that MSc and PhD students from institutions attached to their test facility had had the opportunity to train in a GLP environment as a result of the developed quality management systems. (Quotes: INS2)

Infrastructural improvements at test facilities enhanced the research environment including laboratory, office and shared spaces. Areas of test facilities that were built from scratch or were refurbished included: insecticide testing laboratories, molecular laboratories, insecticide spray rooms, bed net washing areas, insectaries and animal houses. Enhancements included installation of new equipment, improved separation between resistant and non-resistant mosquito strains in insectaries, construction of new facilities to allow new test types (for example, net washing facilities to allow testing of insecticide-treated nets), increased space within existing laboratories, and enhancements to working conditions (e.g. new benching, stools, and wipe-clean tiled surfaces). Installation of new equipment, such as PCR machines, facilitated establishment of new assays and meant that testing of samples could be conducted in-house, reducing the time to obtaining results. Non-laboratory facilities built or refurbished included office spaces, communal break and training areas, facility archives and computer server rooms. For both laboratory and non-laboratory facilities, this enhanced the working environment linked to individuals’ motivation, job satisfaction and pride in their jobs. (Quotes: INS4a)

The research environment was also strengthened through improvements in the procurement processes in some test facilities, and to IT infrastructure across all test facilities. Streamlined procurement processes included the implementation of quality management system practices initiated by the GLP project, in particular in the widespread use of SOPs. This simplified processes and made transfer of work responsibilities when colleagues were absent more seamless. IT infrastructure improvements were relevant across GLP and non-GLP studies, improving processes for accessing and storing study data, managing results in preparation for scientific reports and publications, and improving communication between staff within the test facility through, for example, more widespread use of email and installation of internal telephone systems. (Quotes: INS4b)

### Individual level effects

Whilst the project was focused at the institution level, secondary effects were identified at the individual level. These effects included extensive training, strengthening of career prospects, furtherment of careers, structured working practices and enhanced work motivation.

While areas covered by training programmes varied between test facilities, there was a substantial increase in both breadth and depth in all training programmes. Training examples cited included 24 topics or areas, encompassing training related to QMSs, science specific training, training relating to safety, and business, leadership and life skills training. The training programmes reached staff at all levels of the facility, including non-technical staff such as administrators, drivers, office attendants and gardeners. Training was often specifically tailored to the needs of the test facility staff. (Quotes: IND1)

This training, combined with the practical experience of working in a GLP-compliant laboratory, was highly valued in enhancing career prospects. In all test facilities, staff took on additional responsibilities through, for example, leading on fire safety and organising fire drills or chairing training committees.

Individuals felt an enhanced sense of professionalism and prestige associated with developing and working in a GLP-compliant test facility and seeing the effect of work they had been involved with on changes in vector control policies and practices. This enhanced motivation amongst test facility staff at all levels and technicians and non-scientific staff in particular felt that their work was more structured, meaningful and purposeful following the project (Quotes: IND4). This motivation was enhanced further by an improved working environment following infrastructure improvements, including more working space, air conditioning, and better-quality workstations. (Quotes: IND2)

Examples of career progressions and internal promotions within test facilities were cited across several locations, including promotion of laboratory technicians to laboratory supervisors, and laboratory supervisors to senior management positions. (Quotes: IND3)

### National/international level effects

At the national and international level, identified secondary effects included sharing of best practices within consortia and linked institutions, and the development of regional expertise related to data management and quality assurance.

Test facilities saw increased support from national level institutions, including increased investment in infrastructure. Alongside this, test facilities’ expertise in GLP was recognised at a national level, with the expectation that they would now act as national centres of excellence, both as a model of best practice and as a provider of training in entomology and relevant SOPs. Increased engagement with research outputs at the national decision-making level was anticipated as the next stage of this enhanced relationship with national level institutions, alongside a belief that this would raise policy-makers’ expectations of the test facilities’ performance. (Quotes: NAT1 and NAT2)

At a national and international level, the opportunity to meet and share experiences with the seven collaborating test facilities allowed best practice to be shared throughout the network, although this was not always fully realised as test facilities sought to strike a balance between collaboration and retaining a competitive advantage as a provider of product testing services. For construction and renovation of infrastructure, best practice was shared between test facilities that were geographically close together, because the requirements for buildings were the same and because travelling to these test facilities to see the buildings in person was easier. Data management and quality assurance expertise that was developed in test facilities further along the path to GLP certification, and by individuals associated with these test facilities, was also disseminated through the network. This was done formally through the project network, via training workshops and shared resources such as SOPs, and informally as these individuals acted in consultancy roles both within and outside of the institutions collaborating in the programme. Involvement in this network also raised the profile of individual test facilities, allowing these facilities to attract new studies and collaborators – including both GLP and non-GLP studies. (Quotes: NAT3)

### Non-research capacity strengthening “ripple” effects

Ripple effects of the project beyond research capacity strengthening were widely reported for both individuals and the community surrounding the institutions. At the individual level, these were particularly focused on the transfer of skills developed through training and new practices associated with GLP to home lives. This was particularly true in test facilities that had broad and inclusive training programmes, encompassing topics such as how the test facility was budgeting for GLP and including staff in roles across the test facility such as drivers/cleaners. Here, individuals noted how they had applied time management, organisation, and budgeting skills developed through the GLP project to managing their personal lives and households. (Quotes: IND4).

Effects on communities, which could be described as national level effects, were rooted in often locally sourced solutions to challenges and, in particular, procurement and infrastructure development. By being locally based and finding local solutions, communities around the test facility saw investment in local businesses for consumables, construction materials and construction teams. Also reported was an increase in local employment as new studies were attracted, creating roles such as mosquito collection for experimental hut studies, and improvements in shared infrastructure such as roads. Test facility staff who recognised these effects in the community both took pride in these effects and valued them highly. (Quotes: NAT4).

## Discussion

**Figure 1.**
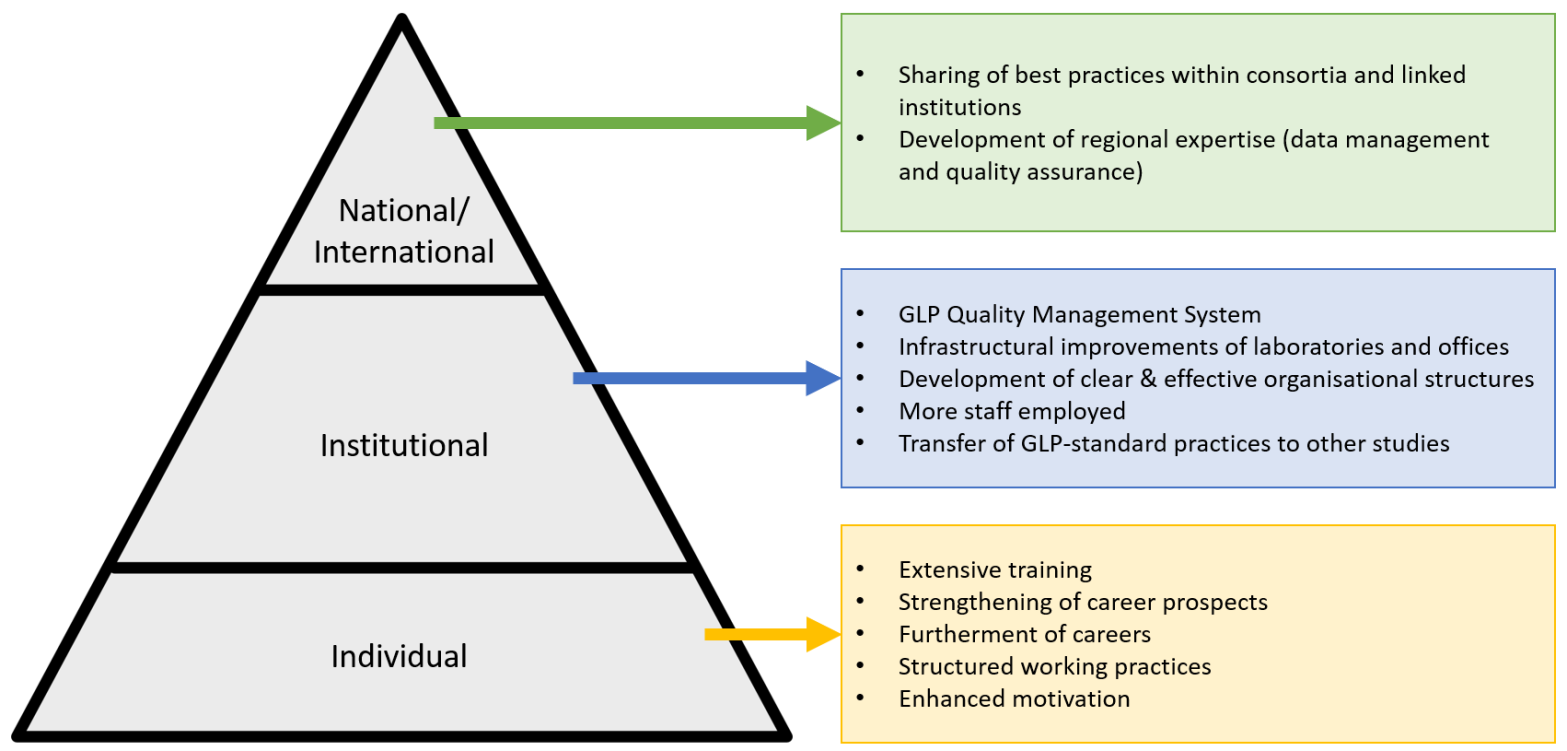
Summary of research capacity effect at the individual, institutional and national/international levels.

Despite a focus on the institutional level, the GLP laboratory capacity strengthening project had effects at each level of the research system – individual, institutional and national/international. These effects are summarised in Figure 1. Primary effects at the institutional level were the development of the GLP quality management system, the central goal of the project, which was achieved through improvements in the infrastructure, research areas and research environment, and including non-research departments such as procurement. This was complimented by enhanced internally delivered training programmes, documentation, human resources processes and organisational structures. Secondary effects at the individual level centred around training, career enhancement, resulting in increased motivation and job satisfaction, for individuals with diverse roles within the test facility. At the national/international level, the secondary effects of the GLP project were increased support and engagement from national level institutions, and the development of opportunities for inter-facility networks and sharing of best practice.

These findings align with factors previously identified for evaluation of research capacity strengthening initiatives ^22^. The findings from this study emphasise that the “research team” included in evaluations of research capacity strengthening should include auxiliary, administrative and technical staff. Therefore, it is imperative that quality training is extended to these roles also, as happened in several test facilities within the GLP project, and that recognition of research leadership/esteem should also encompass recognition of excellence in these roles.

**Figure 2.**
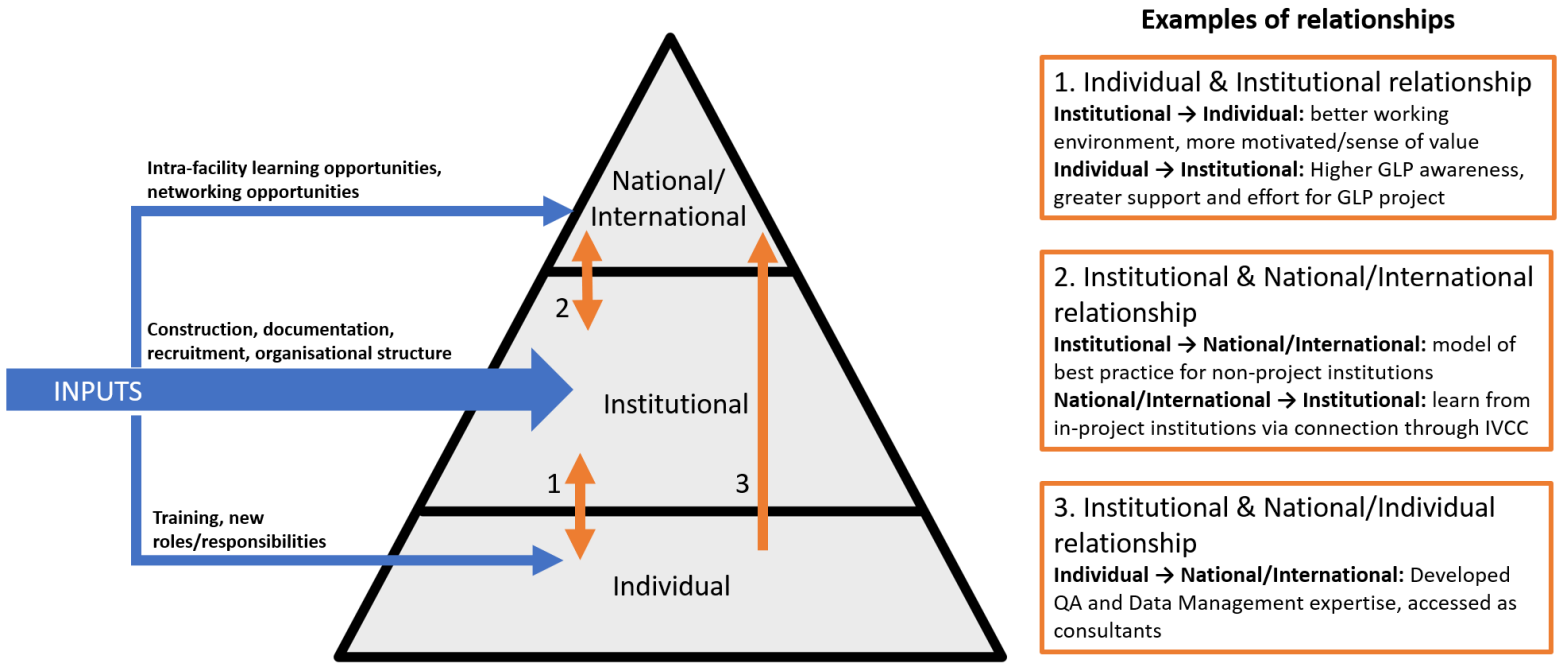
Illustration of inputs for achieving GLP certification at the individual, institutional, and national/international level, and effect relationships between these levels.

The programme was institutionally focused, with the end goal of achieving GLP certification. This, however, required inputs and investment at the individual level (especially training of key individuals, through external workshops or courses, who then went on to implement training in-house or across the network), at the national/international level (for example, by bringing test facilities together to facilitate international networks and collaboration), as well as at the institutional level (an extensive programme of construction and rehabilitation, development of documentation and training programmes, recruitment, and updated organisational structure). A direct effect at these levels was experienced because of this investment, but it also triggered effects across the boundaries between these levels, demonstrating that the three levels within research systems are interconnected ( Figure 2).

This finding supports calls for research capacity strengthening efforts to be explicitly aware of what is happening at all levels and to optimise this effect, even if the described goal is at a single level, in order to plan to optimise these ripple effects ^22– 24^. This may be particularly true for research capacity strengthening initiatives that are targeted at the institutional level, as there is scope for triggering effects across the boundaries with both individual and national/international level, and towards the institution. This also has implications for evaluations of research capacity strengthening initiatives that describe a goal at a single level. In this case, the effects triggered across the boundaries away from the institutional level and jumping directly from the individual to the national/institutional level are effects that contribute to a more broadly strengthened research system without being related to the single-level goal. Nevertheless, these effects are important to capture, both to accurately describe the totality of effects of a programme, but also because the ripple effect at the national/international and individual levels then has an effect of further strengthening at the institutional level.

**Figure 3.**
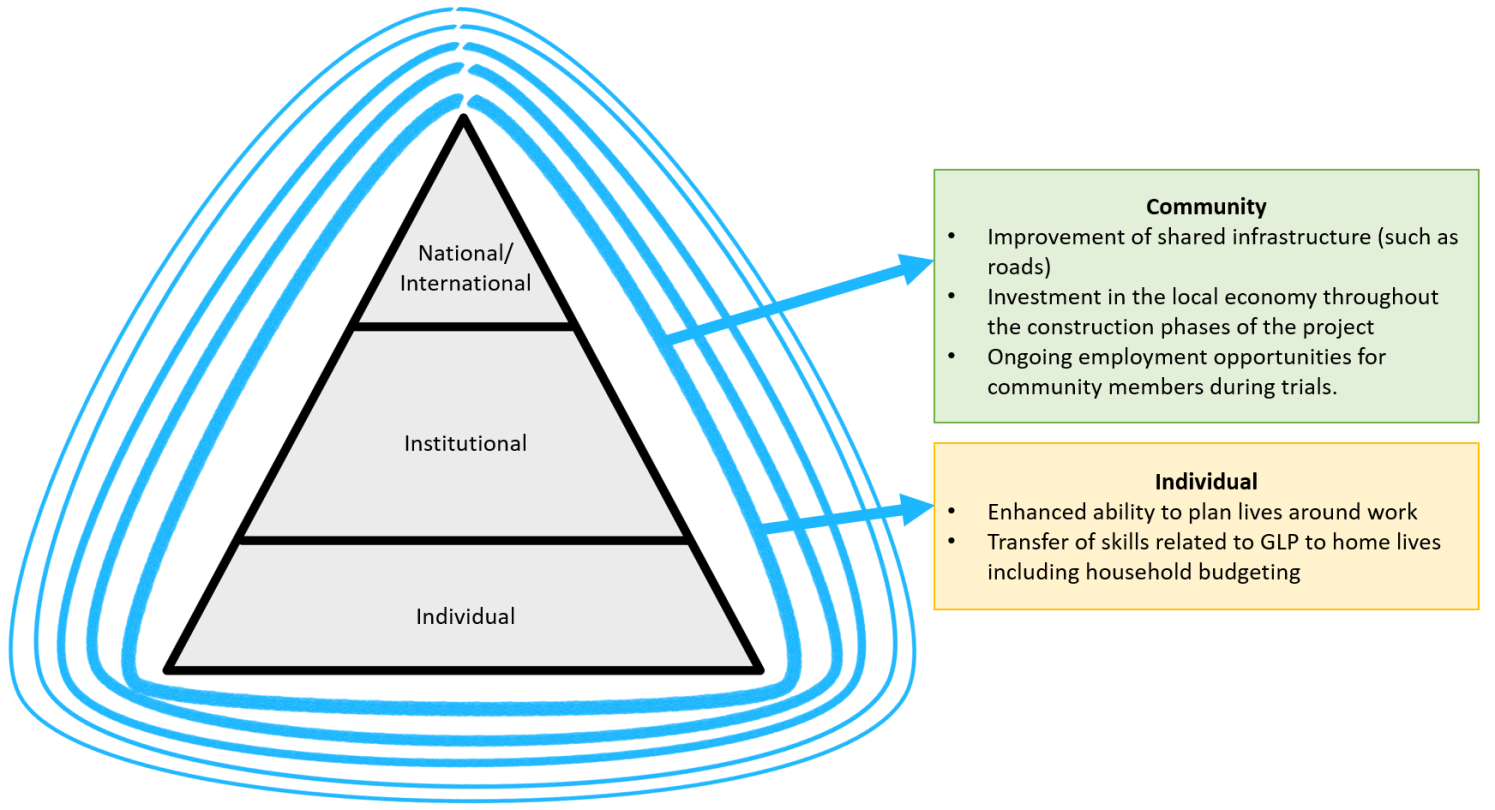
Summary of ripple effects beyond the research system.

Ripple effects were identified beyond the research system, with rich descriptions of how the GLP project was making a wider difference to the lives of the people and communities that surround the test facility ( Figure 3). That these effects were meaningful to those engaged in the GLP project suggests that further exploration of these effects is warranted, and evaluations of similar programmes should expressly plan to capture information about these effects. This is because the ripple effects are an additional source of evidence to engage and motivate individuals in research capacity strengthening projects which, by their nature, have the potential to be challenging and burdensome during implementation.

Together, these findings show that the GLP project acted at and had primary and secondary effects at all three levels of the research system, that the relationship between these levels is complex and interrelated, and that there are ripple effects beyond the research system itself. These findings should, therefore, inform the design and evaluation of similar programmes to:

1. Use the three levels - institutional, individual and national/international - as the foundation for programme development, to promote a holistic approach to programme design, and inform evaluation of effect at each level ^22, 23^;
2. Explicitly plan for and capture information from each level about the interactions with other levels, and capture ripple effects ^22^.

Many indicators for evaluating the outcomes and effect of research capacity strengthening initiatives at all three levels already exist, and these may form the basis of evaluations of similar projects ^7^. Box 1 summarises some suggested areas for consideration when developing evaluations of institutional capacity strengthening projects. For ripple effects in particular a mixed methods or qualitative approach may be beneficial ^25, 26^.

**Box 1. Suggested areas for consideration when developing evaluations of institutional capacity strengthening projects**

- Individual level
  - Broad definition of research team to include auxiliaries, technical staff and administrators, and outcome indicators for training of staff in these roles
  - Broad definition of recognition of leadership to include recognition of proficiency working in a high-quality research system
  - Consider the ripple effect of individual development of transferable life skills

- Institutional level
  - Interrogate the uptake of training programmes to support career development, and the extent to which staff access these programmes.
  - Consider equity of access to these programmes (e.g. gender, role within institution)
  - Consider the extent to which training is integrated into the host institution, with a view to sustainable delivery
  - Consider unintended transferred learning from the research capacity strengthening project to non-research practices across the institution (e.g. to research management support systems) or other research areas
  - Consider the relationship between an improved research environment and staff motivation/job satisfaction

- National/international level
  - Interrogate the extent to which programmes contribute to regional expertise development
  - Consider the ripple effect of investment in communities surrounding the institution

### Strengths and limitations

The strengths of this study are in the diversity of participants involved, capturing the views of staff filling a wide range of roles in five test facilities across three African countries. This approach ensured that effects meaningful to staff in diverse roles were reflected in the findings and offered a voice to staff less often heard within research teams, such as those of technicians and administrators. Furthermore, by using a qualitative approach, this study was able to richly describe the perceived effects of the GLP project and reveal and explain interactions between these effects.

This study is, however, limited by several factors. With a grounding in a specific laboratory capacity strengthening project, caution should be exercised on generalising these findings to all research capacity strengthening projects. Test facilities were at different stages towards GLP certification, with two test facilities having been granted GLP certification to date and this study is unlikely, therefore, to have captured all of the effects of the GLP project. Further effects will likely be identified by staff as the test facilities progress through certification and begin to attract GLP studies from multinational company sponsors. In addition, given the relatively small amount of time specifically dedicated to this question within the interviews, it is likely that additional effects may have been identified given more interview time. Finally, changes had to be made to data collection methods due to the COVID-19 pandemic: the responses at the two test facilities that participated via email and video-call are likely to be more superficial due to reduced opportunities to ask follow-up questions on observations.

## Conclusions

Building research capacity in public health and related fields is essential to the generation of high quality, reliable scientific data. This study, focussing on a project supporting seven test facilities in Africa towards GLP certification, shows that research capacity strengthening interventions for laboratories with a focus on institutional level goals require actions also at individual and national/international levels. The effects of engagement at all three levels towards research capacity strengthening can be amplified by incorporating additional actions at the national/international level, particularly when many institutions are engaged in the same project. This does, however, require that each institution buys into the opportunities for inter-facility learnings for this to collaborative approach to work optimally. Furthermore, there are interactions that happen in both directions across the boundaries between the individual, institutional, and national/international levels, with effects at one level triggering a further effect at another level. These interactions can amplify the effects of an intervention, including research capacity strengthening effects which are the primary objective of such projects. Finally, there are additional “ripple effects” that extend beyond the research system, but that are meaningful to individuals engaged in these projects. The significance of these findings are twofold: firstly, it confirms the interactions between the levels of the research system and, therefore, adds to the evidence that research capacity strengthening projects should plan both to address and to evaluate their effects at all three levels; and secondly, it shows that it is possible to capture the ripple effects of investment in research capacity strengthening and that capturing these effects should be planned for explicitly at the instigation of the project to support further engagement of stakeholders in research capacity strengthening.

## Data availability

### Underlying data

Transcriptions of interviews with facility staff are available from the research group on request (please email ccr@lstmed.ac.uk to request access), on a case by case basis for the purpose of informing further research and on the condition that it will not be published in part or in entirety. They have not been made available as a dataset because they cannot be de-identified without compromising anonymity and the ethical approval conditions for the project stated that only the research team would have access to the data.

### Extended data

Harvard Dataverse: Interview Guide and Information Sheets for: Developing laboratory capacity for Good Laboratory Practice certification: lessons from a Tanzanian insecticide testing facility. https://doi.org/10.7910/DVN/NADZPS ^18^.

This project contains the following extended data:

- Consent Form.docx
- Interview Guide.docx
- Participant information sheet.docx

Data are available under the terms of the Creative Commons Zero "No rights reserved" data waiver (CC0 1.0 Public domain dedication).
