# Ripple effects of research capacity strengthening: a study of the effects of a project to support test facilities in three African countries towards Good Laboratory Practice certification

**DOI:** 10.12688/gatesopenres.13190.2

**Published:** 2021-05-10

**Authors:** Sara Begg, Alexandra Wright, Graham Small, Diabate Abdoulaye, William Kisinza, Benjamin Koudou, Sarah Moore, Franklin Mosha, Constant Edi, Matthew Kirby, Patrick Kija, Robert Malima, Jason Moore, Imelda Bates

**Affiliations:** 1 Liverpool School of Tropical Medicine, Liverpool, L3 5QA, UK; 2 London School of Hygiene & Tropical Medicine, London, WC1E 7HT, UK; 3 Innovative Vector Control Consortium, Liverpool, L3 5QA, UK; 4 Institut de Recherche en Sciences de la Santé, Bobo-Dioulasso, Burkina Faso; 5 National Institute of Medical Research, Amani Centre, Muheza, Tanzania; 6 Centre Suisse de Recherches Scientifques en Côte D’Ivoire, Abidjan, Cote d'Ivoire; 7 Ifakara Health Institute, Ifakara, Tanzania; 8 KCMUCo-PAMVERC, Moshi, Tanzania

**Keywords:** Laboratory, research capacity strengthening, good laboratory practice, insecticide, test facility, quality management system, quality management systems, capacity strengthening

## Abstract

**Background: **Strengthening capacity for public health research is essential to the generation of high-quality, reliable scientific data. This study focuses on a research capacity strengthening project supporting seven test facilities in Africa conducting studies on mosquito vector control products towards Good Laboratory Practice (GLP) certification. It captures the primary effects of the project on each facility’s research capacity, the secondary effects at the individual and institutional level, and the ripple effects that extend beyond the research system. The relationships between effects at different levels are identified and compared to an existing framework for the evaluation of research capacity strengthening initiatives.

**Methods:** To capture the views of individuals engaged in the project at all levels within each facility, a maximum-variation purposive sampling strategy was used. This allowed triangulation between different data sources. Semi-structured interviews were conducted with individuals in three facilities and a combination of email and remote video-call interviews were conducted with individuals at two further facilities.

**Results:** We found that, despite a focus of the GLP certification project at the institutional level, the project had effects also at individual (including enhanced motivation, furtherment of careers) and national/international levels (including development of regional expertise). In addition, we detected ripple effects of the project which extended beyond the research system.

**Conclusion:** This study shows that research capacity strengthening interventions that are focussed on institutional level goals require actions also at individual and national/international levels. The effects of engagement at all three levels can be amplified by collaborative actions at the national/international level. These findings show that research capacity strengthening projects must develop plans that address and evaluate impact at all three levels. Capturing the ripple effects of investment in research capacity strengthening should also be planned for from the beginning of projects to support further engagement of all stakeholders.

## Introduction

Building research capacity in public health and related fields is essential to the generation of robust, innovative and locally relevant scientific data. When research staff are highly skilled and research infrastructure at institutions is strong, the evidence generated by these institutions can inform national policies, support progress towards population health goals and contribute to socioeconomic development ^1– 4^. Research capacity strengthening is increasingly an area of focus for international development and global health partners and funding bodies ^5, 6^. With increasing investment of funds to support research capacity strengthening, there comes an increased need to evaluate the impact of this investment on data quality ^7^. Test facilities are a key component of national research capacity. Attention is commonly focused on clinical diagnostic and research facilities, their role in diagnosis and support in disease and epidemiological surveys ^8^. However, non-clinical and basic science facilities also have key roles to play in global health research ^9^. This can include supporting entomological mapping surveys such as insecticide resistance mapping, generating scientific evidence that can inform the discovery of novel compounds for therapies, development of new products that may have uses in public health, including the control of vectors of diseases, and assessing the safety of these compounds and products before they are used. It is imperative, therefore, that such facilities are included in efforts to build health research capacity, given that not only are they vital for public health, but they also face many of the same challenges and gaps as the more widely researched clinical laboratories ^10, 11^.

This study focuses on a research capacity strengthening project supporting seven test facilities in Africa towards full compliance with Organisation for Economic Co-operation and Development (OECD) principles of Good Laboratory Practice (GLP) ^12^. These test facilities are all engaged in the evaluation of mosquito vector control products, including long-lasting insecticidal nets and indoor residual spraying formulations ^13^. Each test facility consists of an insecticide testing facility (ITF), a molecular biology laboratory, experimental hut sites, an insectary, and animal houses. Data generated by these test facilities inform decision making at a national and international level, as these test facilities have historically conducted laboratory and field efficacy trials on vector control products for evaluation by the WHO Pesticide Evaluation Scheme (WHOPES) ^14^ which supported national programmes and other stakeholders in the selection and safe and judicious use of public health pesticides. With ever-mounting challenges related to increasing insecticide resistance and changes in vector profile and distribution due to climate change, there is a pressing need for innovative vector control products, tools and approaches. To support this, WHO has now transitioned the function for evaluating these products to the WHO Pre-Qualification Team Vector Control (WHO PQT-VC), to align the quality assurance of vector control products with existing prequalification processes within WHO ^15^. Test facilities will now generate data on behalf of companies for the evaluation and prequalified listing of vector control products by WHO PQT-VC, which guides UN agencies, other international organizations and country-level procurement bodies on the procurement of products for malaria management and eradication ^16^. Whilst test facilities are moving towards GLP certification, WHO PQT-VC can inspect data-generating facilities to ensure quality data. However, once sufficient test facilities have been granted GLP certification, WHO PQT-VC will require companies ‘to develop a product dossier which includes data and information to support the safety, efficacy, and quality requirements appropriate to the product type and generated according to Good Laboratory Practices (GLP) and appropriate Quality Management System (QMS)’ ^17^. The conduct of studies compliant with GLP principles will ensure that data generated for product registration purposes are reliable, reproducible and auditable and will be recognised by scientists and regulatory authorities worldwide. Each test facility was supported towards GLP certification by the Innovative Vector Control Consortium (IVCC), with funding from the Bill & Melinda Gates Foundation being used to support the development and implementation of quality management systems, infrastructure improvements, facility inspections to identify and address nonconformances with GLP principles and staff training activities.

Research capacity strengthening has been defined as ‘a process by which individuals, organisations, and society develop the ability to perform [research] functions effectively, efficiently and in a sustainable manner to define objectives and priorities, build sustainable institutions and bring solutions to key national problems’ ^18^. This definition highlights that research capacity strengthening happens at three levels: the individual level, the organisational or institutional level, and the societal or national/international level. In capacity strengthening, initiatives are often focused at one of these three levels ^8, 19^, with programme goals and evaluation of programme success aligning directly with these levels. In this study, the described goal was at the institutional level – developing a QMS compliant with the principles of OECD GLP and being granted GLP certification. Despite an institutional-level goal, the interventions required to implement this system acted at individual, institutional, and national/international levels.

The purpose of this study was to capture both the primary effects of the GLP certification project on each institution’s research capacity, the secondary effects at the individual and institutional level, and any ripple effects beyond the research system. The relationships between effects at different levels are identified. These effects are compared to an existing framework for the evaluation of research capacity strengthening initiatives, to identify new areas for future laboratory capacity strengthening programmes to consider when developing and evaluating their interventions. In addition, we saw ripple effects of the project beyond research capacity strengthening for both individuals within each facility and into the community surrounding them.

## Methods

### Ethical statement

Ethical approval to conduct this research study was obtained from the Liverpool School of Tropical Medicine Research Ethics Committee (approval number 18-041), the National Institute for Medical Research Tanzania (ref NIMR/HQ/R.8c/Vol./I/554), and the Centre Suisse de Recherches Scientifiques en Côte d'Ivoire Institute Review Board (ref 19-549). Institutions taking part remotely (i.e., interviews with members of research staff via Skype/email) provided an institutional approval document in lieu of in-country REC approval, as per point 3c of the LSTM’s Approval Processes for Network and Capacity Strengthening Studies.

Participants were informed about the research using participant information sheets ^20^. Written consent was obtained from each participant prior to undertaking an interview. For individuals in Francophone countries, all consent documentation (participant information sheets and consent forms) was provided in French. In Tanzania, consent documentation was provided optionally in both English and Swahili. All individuals were offered on-site translation into an alternative local language; however, this was not required for any interview participants.

### Setting

This study encompasses five test facilities engaged in the testing of novel vector control products for the purpose of supporting malaria control programmes in Tanzania, Côte D’Ivoire and Burkina Faso. These test facilities are have all received investment and support from IVCC to achieve GLP certification, and are part of a wider programme of support for seven test facilities. Throughout the results and discussion below, findings relate to these five test facilities, although there are references to the benefits of being part of a group of seven institutions. The five test facilities ( Table 1) included in this study encompass a diverse array of contexts. Kilimanjaro Christian Medical University College, Pan-African Malaria Vector Research Consortium (KCMUCo-PAMVERC), Tanzania, provides crucial information on how GLP certification can be achieved, being the first insecticide testing facility in Africa to do so. Comparison between East and West African contexts was facilitated through inclusion of Centre Suisse de Recherches Scientifques en Côte D’Ivoire (CSRS) and Institut de Recherche en Sciences de la Santé (IRSS), Burkina Faso. Comparison between government and non-government test facilities was facilitated through inclusion of National Institute for Medical Research (NIMR) Amani Centre, Tanzania and Ifakara Health Institute (IHI), Tanzania. These contrasting test facilities enhanced our ability to identify both direct and indirect effects of investments in developing a QMS. Generalisability of findings was assessed through using these facilities to compare effects of investment in QMS in a diverse range of contexts, including different national policy contexts and government/non-government supported test facilities.

**Table 1.** Description of participating test facilities and their affiliations.

Test facility	Location	Abbreviation	Affiliations	Studies conducted
Centre Suisse de Recherches Scientifques en Côte D’Ivoire	Abidjan, Côte D’Ivoire	CSRS	CSRS was established in 1951 and is under the dual supervision of the Ministry of Higher Education and Scientific Research (MESRS) in Côte d'Ivoire and the Swiss State Secretariat for Research and Education (SER) via Swiss Tropical and Public Health. CSRS conducts research and training in the fields of biodiversity, food security, environment, and health.	Laboratory, small-scale experimental hut and large-scale community trials on indoor residual spray products, long-lasting insecticidal nets, larvicides and topical repellents
Ifakara Health Institute	Bagamoyo and Ifakara, Tanzania	IHI	IHI was founded in 1956 and is an independent non- profit organisation registered in Tanzania conducting research and training in biomedical & ecological sciences, interventions & clinical trials, health-systems, and policy	Laboratory, small-scale experimental hut and large-scale community trials on indoor residual spray products, long-lasting insecticidal nets, topical repellents, and genetically modified mosquitoes
Institut de Recherche en Sciences de la Santé	Bobo-Dioulasso, Burkina Faso	IRSS	IRSS is an institute of the National Center for Scientific and Technological Research (CNRST) of Burkina Faso. IRSS was created in 1997 to coordinate health related research in Burkina Faso.	Laboratory, small-scale experimental hut and large-scale community trials on indoor residual spray products, long-lasting insecticidal nets and genetically modified mosquitoes
Kilimanjaro Christian Medical University College, Pan-African Malaria Vector Research Consortium	Moshi, Tanzania	KCMUCo- PAMVERC	A malaria research facility established in 2008 via a collaboration between (KCMUCo), Tumaini University, Moshi and the London School of Hygiene and Tropical Medicine (LSHTM), London, UK	Laboratory, small-scale experimental hut and large-scale community trials on indoor residual spray products, long-lasting insecticidal nets and larvicides
National Institute for Medical Research, Amani Centre	Muheza, Tanzania	NIMR Amani Centre	Established in 1949 and operates under the National Institute for Medical Research (NIMR) and conducts vector biology and disease control research	Laboratory, small-scale experimental hut and large-scale community trials on indoor residual spray products, long-lasting insecticidal nets, larvicides and topical repellents

### Sampling

To capture the views of individuals who had exposure to the GLP certification process at all levels of these test facilities, a maximum-variation purposive sampling strategy was used ^21^. This sampling method intentionally seeks to capture a wide range of views, to identify important shared patterns and points of contrast or conflict. For the purpose of this study, the key dimension of variation was role within the test facility, in recognition that this will have determined both which aspects of the GLP certification process individuals were involved with, and the tasks and duties required of them. Sampling included those who hold key roles within a test facility, as determined by a case-study conducted on the first test facility to achieve GLP certification, KCMUCo-PAMVERC ^22^, as well as multiple representatives at each organisational level of the facility. This allowed triangulation between different data sources to determine the trustworthiness of findings. Test facility organograms were used to identify relevant participants, with guidance from stakeholders at IVCC and GLP project managers.

### Data collection and analysis

Semi-structured interviews were conducted with individual staff members involved in the GLP process in three test facilities: KCMUCo-PAMVERC, NIMR Amani Centre, and CSRS. The interview topic guide ^20^ was developed based on previous studies of laboratory capacity strengthening ^8^, with additional questions derived from findings from a case study of the GLP certification process at PAMVERC-KCMUCo ^22^. One overarching question was specifically related to perceived effects of the project. However, due to the semi-structured nature of the interview, interview participants reflected on the effect of the project throughout the interview. Specific questions asked from the topic guide were matched to the roles and responsibilities of the interviewee. Interviews were audio-recorded and transcribed in full. All interviews were conducted in person, in a private room or office, by two researchers, one of whom had a technical understanding of GLP requirements in insecticide testing facilities and the other having systems evaluation experience. Whilst the lead researcher spoke basic French and Swahili, for interview participants who preferred to undertake the interview in a language other than English, a trusted colleague or research student sat in on the interview to aid with translation.

A combination of email and remote video-call interviews were conducted with individual staff members involved in the GLP process at two other test facilities, IRSS and IHI. This was necessitated by restrictions on travel and reduced working hours following the COVID-19 pandemic, which resulted in significant disruption from March 2019. The overarching questions asked in these interviews were retained from the semi-structured interview guide used for in-person interviews. Follow-up questions, where relevant, were conducted via video-call or email.

A framework analysis ^23^ was used to identify themes emerging from the interview transcripts following the five-step process of familiarization, identification of thematic framework, indexing, charting and mapping/interpretation. The framework identified was the Research Capacity Strengthening evaluation framework developed by Khisa *et al.*, from African Population and Health Research Center, Nairobi, Kenya and Centre for Capacity Research, Liverpool School of Tropical Medicine, UK ^24^. This framework delineates the identified and envisioned effect of research capacity strengthening initiatives at the individual, institutional, and national/international level, developed from a review of the research capacity strengthening literature and refined in consultation with research capacity strengthening funders, implementers, managers and evaluators ( Table 2).

**Table 2.** Framework for evaluating Research Capacity Strengthening from Khisa *et al.*, 2019 ^24^.

Individual level	Institutional level	National/international level
Provision and quality of training for the research team	Career pathways for the research team	National: research councils/research productivity
Recognition of research leadership/esteem	Sustainable provision of appropriate, high quality training	International: networks/ collaborations
Career trajectory	Nationally/internationally competitive research and grants	Research effect and user engagement
	Research environment – finance, library, IT, labs etc	

This framework’s conceptualisation of research capacity strengthening initiatives happening at three levels, individual, institutional, and national/international, is rooted in the understanding that while these three levels have different foci, they are interconnected, with interventions at one level both influencing and being influenced by factors at other levels ^25^. Broadly speaking, at the individual level the focus is typically on the development of researchers and teams, at the institutional level the focus is on development of systems and processes within university departments or other organizations/institutions, and at the national/international level the focus is on influencing structural factors including policy, regulation and research networks ^25, 26^.

Following familiarisation with the interview data, further themes were identified and incorporated into the framework, while retaining the individual, institution, and societal level structure. All interview transcripts were indexed using NVivo software version 11 (QSR International).

## Results

A total of 65 members of staff from five test facilities participated in this study. 66 were approached to take part, with one declining to take part. Of these staff, 16 were laboratory/insectary technicians or attendants, 17 were from non-scientific administration/information technology positions, 22 were from scientific middle-management positions, and 11 were from scientific senior management positions. 49 were male and 16 were female. Anonymised identifiers have been used for quotes from transcripts, highlighting the role of the interview participant but not the test facility they are connected to. These are presented in supplementary materials (Effects of GLP project.tab) and referenced by section in the text. Table 3 summarises themes as they relate to the individual, institutional and national/international levels, and two illustrative quotes for each theme are presented. Where relevant, illustrative quotes are from individuals in differing roles.

**Table 3.** Themes identified through framework analysis of semi-structured interviews. Illustrative quotes related to each theme are provided, with examples from individuals in differing roles, where relevant. More extensive illustrative examples related to each theme are available in supplementary materials.

Individual Level	Illustrative quote 1	Illustrative quote 2
Provision and quality of training for the research team (IND1)	We had some training from [IVCC member of staff 1] on the development of SOPs... I also had training on GLP which was conducted in Moshi. That was the first one. It was big because it combined different sites… It exposed us to this process, why do we need to do it, why should we change our attitudes towards what we are doing, what's the value of it. *Laboratory Supervisor*	We initially conducted external training for all staff on general GLP principles from [IVCC member of staff 2] which was useful as it put all evels of staff on a strong foundation. *Test Facility Manager*
Recognition of research leadership/ esteem (IND2)	I think the team as well would be happy to see the products which have been evaluated here and found to be effective as seen in the market… I’m saying this because I’ve been involved in evaluating a number of these products. When I go out there even in other missions and I found those products in the market, it's a great feeling and I can tell the story. *GLP Project Coordinator*	Yes, because we are now professional. Professional in everything-- when you do something and you see the results you think, "Yes, I've done it." It's a feeling of professionalism. *Technician*
Career trajectory (IND3)	I must admit that in the government system, we don't train these people that much. Here, the system was good for scientists and technicians but not for supportive staff. With GLP, at least they're now considered. They get training on what to do, which is very good for their career as well. *Research Scientist*	Yes, for instance, it's good in your resume if you're working in a place which has accreditation, it’s a big plus. It means you're fit to work there. *Technician*
Structured work practices (IND4)	Because of how GLP wants you to work, it helps you to be creative. To be creative, so that you can do what you are supposed to do. That has helped me a lot. To manage the work, and to manage the people you're working with. *Laboratory Supervisor*	I think I actually learned a better way of how to maintain or how to keep track of what I'm doing. This has actually been a good way from you actually know like everything where everything is and if I want to remember something, I don't have to actually guess about it. I have a log of everything that I have done. *Administrator*
Transfer of organisation skills to home (IND5)	When we talk about GLP, the best thing it gave us is a way to govern your life. Because apart from being here working with GLP, it helps also us to know that in life you have to follow some guideline, and you have to do things followed by some rules. Personally, I have found this very instructive. *GLP Manager*	GLP also is teaching us how to be punctual. Not punctual only in the working place, but in your family. GLP is helping us to save, in saving, in budgeting. That is something which somebody can't see, so is to me is indirect benefit. *Laboratory Supervisor*
**Institutional level**		
Career pathways for the research team (INS1)	There is what we call performance appraisal. Normally we appraise people quarterly. I do find things they are moving more easily, because people have to fill the forms. When you see the comments from the head of department, you find heads of department are doing their part. Even the staff are doing their part. I find it has made my work easy. *Administrator*	People, I think, generally want to be trained more. Maybe that desire always existed but there wasn't a channel for people to voice that and now there is. We have appraisals, we have the training committee. *Study Director*
Sustainable provision of appropriate, high quality training (INS2)	Internally, there have been trainings on GLP several times. Those trainings concerned general aspects of GLP and specific aspects such as writing SOPs and their use. Those internal trainings were done at our institution by the quality manager and the supervisors. *Laboratory Supervisor*	Also, it advertises the college as well. We have been training some the college students at the Master's level, and they've been attached here for their Master's as well. *GLP Project Coordinator*
Nationally/ internationally competitive research and grants (INS3)	That is one of the success that we had. Also, the other issue is that we managed to attract some clients, looking for our technical support and the evaluation of their products. For instance, for the phase one evaluation of products-- Since the inception of the workshop in Liverpool, we had about three-phase one studies. *Test Facility Manager*	We can give data that is trustworthy since it is collected in a defined standard and by using well maintained and validated equipment. Most of all, the output of good quality data from research is for the benefit of the whole community i.e. when we say a certain product is efficacious then it’s really so. This then means protection of the whole public. *Quality Assurance Manager*
Research environment – infrastructure (INS4a)	I think that before when you worked in different projects the infrastructure was sometimes not adapted to the entomology. Now the infrastructure is there, when you go to the insectarium and to the lab you see there is new materials. There are meeting rooms, and the archive office. Also, in [Field Site] there is a new building. We see that there is some evolution. *Laboratory Supervisor*	You can see now the condition of working for every one of us-- scientists, the technicians, the assistants-- has been very drastically improved. Even there at the [Field Site], the rooms now, they are very comfortable for the people who are sleeping; very comfortable. *Laboratory Supervisor*
Research environment (b) – IT, human resources, procurement (INS4b)	I'll actually say the computers are not really that expensive, but the major part is having the main primary place where you can actually do everything. For us, it made it easier for us to control most of our research activities. We have created easier, formal ways to access things. I think with the findings and everything that we've got it, it should help a lot with putting up research. *Data Manager*	Each service had its way of doing their work. With the SOPs, they guide them to do the things they need to do for each task. For instance, we had processes in accounts, but they have a lot of papers, documents that they have to validate, if it is to procure some material, some things, it will come to their site, they would just validate it. But with the SOP, you know what to validate, what you shouldn't validate. So, it's kind of a guiding that help them, each service interacts more smoothly. *Administrator*
Structured working practices (INS5)	First, we have learned to be accountable. I myself I have learned to value every [national currency] that we get; to get value for money. The way we used to work before GLP is quite different. GLP money has done more than what we expected it could do, after starting, working with the seriousness, making sure that we get standard material, things like that. *Laboratory Supervisor*	It helped the management and technical team to focus efforts in a more structured way for general working practices and enabled full traceability of test items and experiments. *Test Facility Manager*
**National/** **International level**		
National: research councils/research productivity (NAT1)	For example, we have a centre for medical entomology and veterinary they really want us to train their students to use our lab and there is the National Institute for Public hygiene they're doing a lot of entomology survey and they want us to process the sample. I told you about the PMI project, so we're using the lab to process the mosquito sample we collect. *Director*	You can see that GLP becomes very interesting. It becomes a centre of excellence for training in this area. These government universities prefer to go to a government institution or centre. *Laboratory Supervisor*
International: networks/ collaborations (NAT2)	We visited [Collaborating Test Facility] to see how they have- how they have gone, how far have they gone, and what challenges they did. We learned from them, actually. *Laboratory Supervisor*	As a network with other institutions, like we have local institution like [Collaborating Test Facility] who have already been accredited, and because we have collaboration with [Vector Control Network], we are working with them. We have the network from West Africa and also there are other people from [UK Institution] actually because our long collaborators. We get a lot of technical support from different people in terms of advice and what to do. And because of their advice we can make sure the site is well equipped and meets the requirements for GLP. *Test Facility Manager*
Research impact and user engagement (NAT3)	As [Test Facility], I think the main benefit is to provide - we normally say that if you don’t have data, you don’t have the right to speak - to provide data which will actually influence the policy change for vector control in the country. *GLP Project Coordinator*	The Ministry of Health are anxious because the [Umbrella Institution] is the technical arm of the Ministry of Health, so they are looking forward to make sure they have a very competent technical arm that can provide good advice related to vector control and evaluation of new vector control tools. *Test Facility Manger*
Effect of investment in surrounding community (NAT4)	Those people, they really liked the project. The same people from the environment were very happy to be involved in part of the work. They said, “It's useful for us. Our kids stay there. They live there.” It was easy to recruit people for working there in the site. Very easy. *Laboratory Supervisor*	The renovation activity which has been done here benefitted the entire community of [Region]. The businesspeople who we are working with, the retailer shops where we were getting materials for the construction, the people here who worked here, we all benefited from this. That money went to them, actually, we had to buy materials and to construct. We had good relationship with them during the entire process. Even there at the villages, the way we renovated our buildings, the way we are taking care of the road to reach there… because of the projects that keeps the volunteers, they get some incentives, things like that, it has been very nice. They got the benefit out of it. *GLP Project Coordinator*

From the interviews, the research capacity strengthening effect of the programme at the all three level was consistently identified, despite the project’s focus on the institutional level. At the individual level these effects were related to the training delivered as part of the GLP project, but there was also a positive relationship between the institutional level effects of improved research environment (both physical and administrative) and individual level motivation and job satisfaction. Further institutional level effects encompass sustainable provision of training, and enhanced capacity to deliver competitive research, i.e. GLP-compliant studies. At the national/international level, networks between institutions were developed, which further strengthened individual test facilities (institutions) as inter-facility learning was made possible.

### Individual level effects

Whilst the project was focused on the institution level, important effects were identified at the individual level. These included extensive training, strengthening of career prospects, furtherment of careers, structured working practices and enhanced work motivation.

There was a substantial increase in both breadth and depth in all training programmes. Training examples cited included 24 topics or areas, encompassing training related to QMSs, science specific training, training relating to safety, and business, leadership and life skills training. Training reached staff at all levels of the facility, including non-technical staff such as administrators, drivers, office attendants and gardeners, and was often specifically tailored to the needs of the test facility staff (Quotes: IND1). This training, combined with the practical experience of working in a GLP-compliant laboratory, was highly valued as enhancing career prospects. In all test facilities, staff took on additional responsibilities through, for example, leading on fire safety or chairing training committees.

Individuals felt an enhanced sense of professionalism and prestige associated with developing and working in a GLP-compliant test facility. This was reflected in seeing changes in vector control policies and practices informed by the work they had been involved with. This enhanced motivation amongst test facility staff at all levels, and technicians and non-scientific staff in particular felt that their work was more structured, meaningful and purposeful (Quotes: IND4). This motivation was enhanced further by an improved working environment following infrastructure improvements, including more working space, air conditioning, and better-quality workstations (Quotes: IND2).

Together, these effects positively impacted on career progression for individuals. Examples of career progressions and internal promotions within test facilities were cited across several locations, including promotion of laboratory technicians to laboratory supervisors, and laboratory supervisors to senior management positions. (Quotes: IND3)

### Institutional level effects

At the institutional level, the GLP quality management system, infrastructural improvements of laboratories and offices, development of clearer and more effective organisational structures, more staff employed, and the transfer of GLP-standard practices to other studies were all identified as research capacity strengthening effects.

The development of a GLP-compliant quality management system and, at some test facilities, the achievement of GLP certification, is a clear outcome of the work undertaken through the IVCC project. Of the five test facilities included in this study, one has achieved GLP certification to date, and three have submitted their application for GLP certification to SANAS. As a result of support towards GLP compliance and certification, these test facilities were able to deliver national/internationally competitive research, with data meeting international standards. This effect extended also to non-GLP studies conducted at these test facilities, as best practice from GLP studies was applied to non-GLP studies by both scientists involved in the GLP project and other scientists within the institution, particularly with respect to study documentation and use of Standard Operating Procedures (SOPs). Thus, the overall quality of data generated at these test facilities was enhanced. (Quotes: INS3) Test facilities also identified broader effects on working practices, resulting from the implementation of GLP standards. In particular, increased structure in working practices resulting in benefits including cost savings on reagents, more effective problem solving, and better organisation of work throughout the test facility. (Quotes: INS5)

Career pathways were enhanced by strengthening the processes, policies, and documentation that surrounded organisational structure and human resources. Clearer organisational structures facilitated communication between individuals in different departments and at different levels within the test facility. This was supported through SOPs for regular, documented human resource support including appraisals and Curriculum Vitae review. Together, these had an additional effect on individuals’ sense of place and therefore, sense of value within the test facility. In some test facilities, new structures were put in place for requesting training for career development, and staff were adequately empowered to take up these opportunities. Across test facilities, but particularly in those that had achieved GLP certification, there were more job opportunities at the institution, with studies and investment attracted to the test facility. (Quotes: INS1)

In-house training programmes were developed and delivered across test facilities including general training in GLP awareness, Quality Assurance, training in SOPs, Health and Safety/Fire training, archiving training, leadership training, and computer system validation and usage. Training programmes were often developed by test facility staff following attendance at externally delivered training courses. Implementation of training was overseen by staff in a range of roles, as staff at all levels took on additional responsibilities. Test facility management noted that MSc and PhD students from institutions attached to their test facility had had the opportunity to train in a GLP environment, and this was a point of prestige for the institution. (Quotes: INS2)

Infrastructural improvements enhanced the research environment including laboratory, office and shared spaces. Areas that were built from scratch or were refurbished included: insecticide testing laboratories, molecular laboratories, insecticide spray rooms, bed net washing areas, insectaries and animal houses. Enhancements included installation of new equipment, improved separation between resistant and non-resistant mosquito strains in insectaries, construction of new facilities to allow new test types, increased space within existing laboratories, and enhancements to working conditions (e.g. new benching, stools, and wipe-clean tiled surfaces). Installation of new equipment, such as PCR machines, facilitated establishment of new assays and meant that testing of samples could be conducted in-house, reducing the time to obtaining results. Non-laboratory facilities built or refurbished included office spaces, communal break and training areas, facility archives and computer server rooms. For both laboratory and non-laboratory facilities, this enhanced the working environment, linked to individuals’ motivation, job satisfaction and pride in their jobs. (Quotes: INS4a)

The research environment was also strengthened through improvements in the procurement processes in some test facilities, and to IT infrastructure across all test facilities. Streamlined procurement processes included the implementation of quality management system practices initiated by the GLP project, in particular in the widespread use of SOPs. This simplified processes and made transfer of work responsibilities more seamless. IT infrastructure improvements were relevant across GLP and non-GLP studies, improving processes for accessing and storing study data, managing results in preparation for scientific reports and publications, and improving communication between staff within the test facility through more widespread use of email and installation of internal telephone systems. (Quotes: INS4b)

### National/international level effects

At the national and international level, identified effects included sharing of best practices within consortia and linked institutions, and the development of regional expertise related to data management and quality assurance.

Test facilities saw increased support from national level institutions, including increased investment in infrastructure. This was often coupled with the expectation that they would now act as national centres of excellence, both as a model of best practice and as a provider of training in entomology and relevant SOPs. Increased engagement with research outputs at the national decision-making level was anticipated as the next stage of this enhanced relationship with national level institutions, alongside a belief that this would raise policymakers’ expectations of the test facilities’ performance. (Quotes: NAT1 and NAT2)

At a national and international level, the opportunity to meet and share experiences with collaborating test facilities allowed best practice to be shared throughout the network, although this was not always fully realised as test facilities sought to strike a balance between collaboration and retaining a competitive advantage as a provider of product testing services. For construction and renovation of infrastructure, best practice was shared between test facilities that were geographically close together, because the requirements for buildings were the same and because travelling to these test facilities to see the buildings in person was easier. Data management and quality assurance expertise that was developed by individuals in test facilities further along the path to GLP certification was also disseminated through the network. This was done formally through the project network, via training workshops and shared resources such as SOPs, and informally as these individuals acted in consultancy roles both within and outside of the institutions collaborating in the programme. Involvement in this network also raised the profile of individual test facilities, allowing these facilities to attract new studies and collaborators – including both GLP and non-GLP studies. (Quotes: NAT3)

### Non-research capacity strengthening “ripple” effects

Ripple effects of the project beyond research capacity strengthening were widely reported for both individuals and the community surrounding the institutions. At the individual level, these were particularly focused on the transfer of skills developed through training and new practices associated with GLP to home lives. This was particularly true in test facilities that had broad and inclusive training programmes. Here, individuals noted how they had applied time management, organisation, and budgeting skills developed through the GLP project to managing their personal lives and households (Quotes: IND4).

Effects on communities surrounding the institution were rooted in often locally sourced solutions to challenges and, in particular, procurement and infrastructure development. By being locally based and finding local solutions, communities around the test facility saw investment in local businesses for consumables, construction materials and construction teams. Also reported was an increase in local employment as new studies were attracted, creating roles such as mosquito collection for experimental hut studies, and improvements in shared infrastructure such as roads. Test facility staff who recognised these effects in the community both took pride in these effects and valued them highly. (Quotes: NAT4).

## Discussion

**Figure 1.**
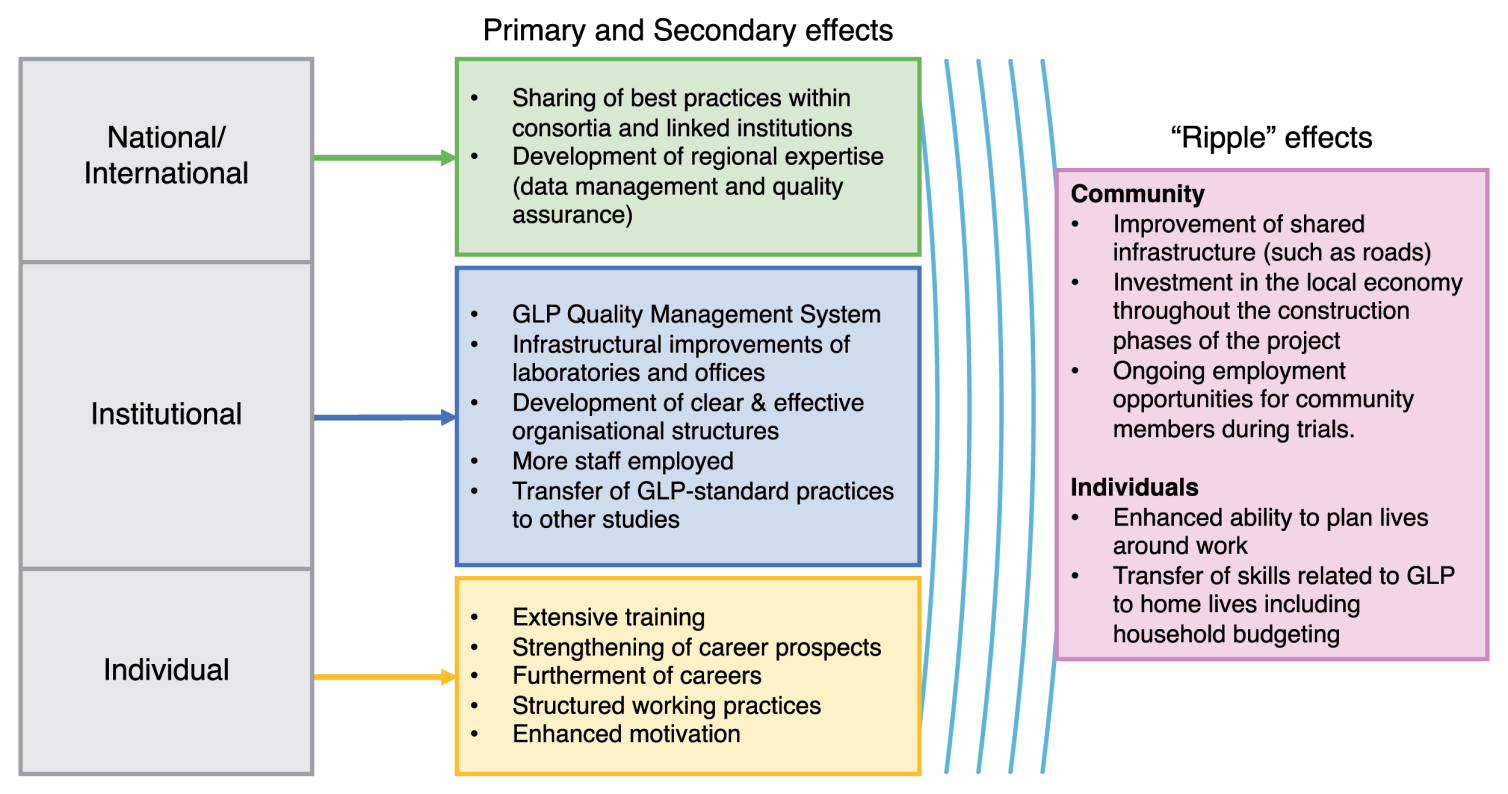
Summary of research capacity effect at the individual, institutional and national/international levels.

Despite a focus on the institutional level, the GLP laboratory capacity strengthening project had effects at each level of the research system – individual, institutional and national/international. These effects are summarised in Figure 1. These findings align with factors previously identified for evaluation of research capacity strengthening initiatives ^24^. The findings from this study emphasise that, particularly at the individual level but also at the institutional level, the “research team” included in evaluations of research capacity strengthening should include auxiliary, administrative and technical staff. These roles are often neglected in RCS evaluations but are vital for implementation of quality research. It is also imperative that quality training is extended to these roles, as happened in several test facilities within the GLP project. Recognition of research leadership and esteem should not be limited to evaluation of outputs of research scientists in middle and senior management roles but should also encompass recognition of excellence in administrative and technical roles.

**Figure 2.**
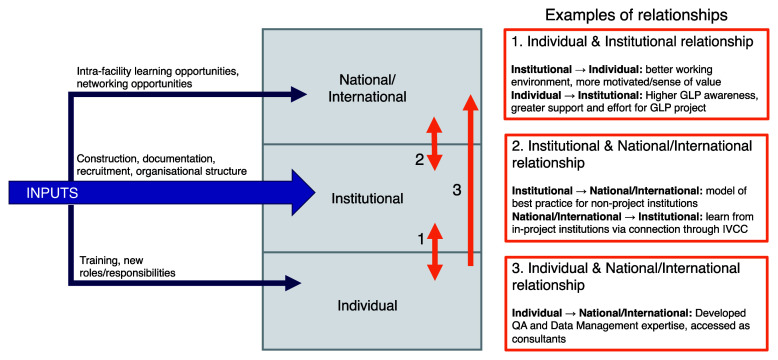
Illustration of inputs for achieving GLP certification at the individual, institutional, and national/international level, and effect relationships between these levels.

The programme was institutionally focused, with the end goal of achieving GLP certification. This, however, required inputs and investment at the individual level (especially external training of key individuals, who then went on to implement training in-house or across the network), at the national/international level (for example, by bringing test facilities together to facilitate international networks and collaboration), as well as at the institutional level. A direct effect at these levels was experienced because of this investment, but it also triggered effects across the boundaries between these levels, demonstrating that the three levels within research systems are interconnected ( Figure 2), and reflecting findings from previous evaluations of individual level initiatives that showed positive secondary effects on national and international collaboration ^11^.

This finding supports calls for research capacity strengthening efforts to be explicitly aware of what is happening at all levels and to optimise this effect, even if the described goal is at a single level, in order to plan to optimise these secondary and ripple effects ^24, 26, 27^. This may be particularly true for research capacity strengthening initiatives that are targeted at the institutional level, as there is scope for triggering effects across the boundaries with both individual and national/international level, and towards the institution. This also has implications for evaluations of research capacity strengthening initiatives that describe a goal at a single level. In this case, the effects triggered across the boundaries away from the institutional level and jumping directly from the individual to the national/institutional level are effects that contribute to a more broadly strengthened research system without being related to the single-level goal. Nevertheless, these effects are important to capture, both to accurately describe the total effect of a programme, but also because strengthening at the national/international and individual levels then has an effect of further strengthening at the institutional level.

Ripple effects were identified beyond the research system, with rich descriptions of how the GLP project was making a wider difference to the lives of the people and communities that surround the test facility ( Figure 1). Unexpected effects arising from research capacity strengthening initiatives have been previously identified, particularly in the development of transferable skills ^11, 28^. The findings presented here highlight beneficial effects for communities close to the testing sites which were meaningful to those engaged in the GLP project. Explaining these benefits to those involved in research capacity strengthening projects may help to engage and motivate them during difficult times on the project. Future research could further explore these effects, to better understand how they arise, to what extent they are attributable to the research capacity strengthening efforts, and the impact of these effects on both individuals and communities. 

Together, these findings show that the GLP project acted at and had primary and secondary effects at all three levels of the research system, that the relationship between these levels is complex and interrelated, and that there are ripple effects beyond the research system itself. These findings should, therefore, inform the design and evaluation of similar programmes to:

1. Use the three levels - institutional, individual and national/international - as the foundation for programme development, to promote a holistic approach to programme design, and inform evaluation of effect at each level ^24, 26^;
2. Explicitly plan for and capture information from each level about the interactions with other levels, and capture ripple effects ^24^.

Many indicators for evaluating the outcomes and effect of research capacity strengthening initiatives at all three levels already exist, and these may form the basis of evaluations of similar projects ^7^. Box 1 summarises some suggested areas for consideration when developing evaluations of institutional capacity strengthening projects. For ripple effects in particular a mixed methods or qualitative approach may be beneficial ^29, 30^.

**Box 1. Suggested areas for consideration when developing evaluations of institutional capacity strengthening projects**

- Individual level
  - Broad definition of research team to include auxiliaries, technical staff and administrators, and outcome indicators for training of staff in these roles
  - Broad definition of recognition of leadership to include recognition of proficiency working in a high-quality research system
  - Consider the ripple effect of individual development of transferable skills

- Institutional level
  - Interrogate the uptake of training programmes to support career development, and the extent to which staff access these programmes.
  - Consider equity of access to these programmes (e.g. gender, role within institution)
  - Consider the extent to which training is integrated into the host institution, with a view to sustainable delivery
  - Consider unintended transferred learning from the research capacity strengthening project to non-research practices across the institution (e.g. to research management support systems) or other research areas
  - Consider the relationship between an improved research environment and staff motivation/job satisfaction

- National/international level
  - Interrogate the extent to which programmes contribute to regional expertise development
  - Consider the ripple effect of investment in communities surrounding the institution

### Strengths and limitations

The strengths of this study are in the diversity of participants involved, capturing the views of staff filling a wide range of roles in five test facilities across three African countries. This approach ensured that effects meaningful to staff in diverse roles were reflected in the findings and offered a voice to staff less often heard within research teams, such as those of technicians and administrators. Furthermore, by using a qualitative approach, this study was able to richly describe the perceived effects of the GLP project and reveal and explain interactions between these effects.

This study is, however, limited by several factors. As no quantitative data is included in this study, numerical measures of change resulting from the GLP project are not possible. Instead, the study relies on the subjective experiences and opinions of individuals involved in the GLP project. With a grounding in a specific laboratory capacity strengthening project, caution should be exercised on generalising these findings to all research capacity strengthening projects. Test facilities were at different stages towards GLP certification and this study is unlikely, therefore, to have captured all of the effects of the GLP project. Further effects will likely be identified by staff as the test facilities progress through certification and begin to attract GLP studies. In addition, given the relatively small amount of time specifically dedicated to this question within interviews, it is likely that additional effects may have been identified given more interview time. Finally, changes had to be made to data collection methods due to the COVID-19 pandemic: the responses at the two test facilities that participated via email and video-call are likely to be more superficial due to reduced opportunities to ask follow-up questions on observations.

## Conclusions

Building research capacity in public health and related fields is essential to the generation of high quality, reliable scientific data. This study, focussing on a project supporting seven test facilities in Africa towards GLP certification, shows that research capacity strengthening interventions for laboratories with a focus on institutional level goals also require actions at individual and national/international levels. Furthermore, there are interactions that happen in both directions across the boundaries between the individual, institutional, and national/international levels, with effects at one level triggering a further effect at another level. These interactions can amplify the effects of an intervention, including research capacity strengthening effects which are the primary objective of such projects. Finally, there are additional “ripple effects” that extend beyond the research system, but that are meaningful to individuals engaged in these projects. The significance of these findings are twofold: firstly, it confirms the interactions between the levels of the research system and, therefore, adds to the evidence that research capacity strengthening projects should plan both to address and to evaluate their effects at all three levels; and secondly, it shows that it is possible to capture secondary and ripple effects of investment in research capacity strengthening and that capturing these effects should be planned for explicitly at the instigation of the project to support further engagement of stakeholders in research capacity strengthening.

## Data availability

### Underlying data

Transcriptions of interviews with facility staff are available from the research group on request (please email ccr@lstmed.ac.uk to request access), on a case by case basis for the purpose of informing further research and on the condition that it will not be published in part or in entirety. They have not been made available as a dataset because they cannot be de-identified without compromising anonymity and the ethical approval conditions for the project stated that only the research team would have access to the data.

### Extended data

"Illustrative Quotes, Interview Guide and Information Sheets for: Developing laboratory capacity for Good Laboratory Practice certification: lessons from a Tanzanian insecticide testing facility", https://doi.org/10.7910/DVN/NADZPS, Harvard Dataverse, V2 ^20^.

This project contains the following extended data:

- Effects of GLP project.tab
- Consent Form.docx
- Interview Guide.docx
- Participant information sheet.docx

Data are available under the terms of the Creative Commons Zero "No rights reserved" data waiver (CC0 1.0 Public domain dedication).
