# Supplementary material for: Ripple effects of research capacity strengthening: a study of the effects of a project to support test facilities in three African countries towards Good Laboratory Practice certification
Source: Gates Open Res. 2021 May 10;4:175. Originally published 2020 Nov 27. [Version 2] doi: 10.12688/gatesopenres.13190.2 (PMC8167331; doi:10.12688/gatesopenres.13190.2)
Supplement: Peer review history

# Peer review: Reviewer response for version 2

**DOI:** 10.21956/gatesopenres.14497.r30986

**Published:** 2021-08-24

**Authors:** Tonderai Mapako

**Affiliations:** 1 National Blood Service Zimbabwe (NBSZ), Harare, Zimbabwe

I find the article informative and helpful in enhancing the research capacities in similar settings. This is similar to previous work for blood services setting conducted by this research and co-workers (T Mapako et al).

 Just a few issues to take note of:

1. Typo's: e.g. page 4 of 25, section on Setting. Delete the word "are" in the statement "The test facilities **are **have all.."
2. On quotes, avoid repetition of text and tables. I like the Figures summary and they condense the information. Equally text information related to the figures may be minimised to enhance and focus the article.
3. Just to hear the authors view: Given that the qualitative aspects was not feasible for this study, I was just wondering on the choice of the words "ripple effects ..." in the preferred study title. Whilst I appreciate the intention of the messaging not sure whether an alternative expression would not have been more appropriate as this is assessing "perspectives" "opinions" etc without necessarily assessing the "ripple effects" which are self-reported in this context. I hold the view that the use of work "ripple effects" is more strong that what the "study design" seems to accommodate. Will be happy to hear Investigators opinion on this.

Is the work clearly and accurately presented and does it cite the current literature?

Yes

If applicable, is the statistical analysis and its interpretation appropriate?

Yes

Are all the source data underlying the results available to ensure full reproducibility?

Yes

Is the study design appropriate and is the work technically sound?

Yes

Are the conclusions drawn adequately supported by the results?

Yes

Are sufficient details of methods and analysis provided to allow replication by others?

Yes

Reviewer Expertise:

Biostatistician by profession. Research capacity building, research ethics, strategic management and business models

I confirm that I have read this submission and believe that I have an appropriate level of expertise to confirm that it is of an acceptable scientific standard.

## References

[1] : A systematic approach for reviewing research capacity within Zimbabwe's national blood service. *ISBT Science Series*.2016;11(2) : 10.1111/voxs.12276110-11710.1111/voxs.12276

# Peer review: Reviewer response for version 2

**DOI:** 10.21956/gatesopenres.14497.r30643

**Published:** 2021-05-28

**Authors:** Michael Käser

**Affiliations:** 1 Swiss Tropical and Public Health Institute, Basel, Switzerland

After the revisions taken, the study has gained clarity and has become more concise in its outline and appearance, the figures more sound, and the manuscript should be made available to the research community, in particular the one involved in (global) RCS.

 Still, the write-up of the manuscript would benefit from some continued minor revisions to gain clearness and improved readability, as described in the following:

**Results presentation and clarity**

- Although the authors’ answer mentions that repetitions (i.e. on the ripple effect) are being removed, there are still too many of them. I assess it is possible to still shorten the text by making it more concise and better readable. Still, it is not surprising that such a project has additional positive side effects so that the “ripple effects” should not be overemphasized which I feel still are.
- The reader still does not spot easily that only 5 centres are being investigated on if not reading the revision comments before (“Clarified throughout that effects described in this study are in the 5 test facilities involved in the study (described in Table 1) and highlighted where national/international level effects arose from the wider group of 7 test facilities”) True that mention is at the beginning of the results but should be mentioned at least towards the end of the introduction and more visibly in the methods section.
- The reduction and colour code clarification throughout all tables and figures is refreshing.
- I welcome the shift of statements into the supplementary material;
- And yet Table 3 is still difficult to digest, in particular since the mention “Selected two illustrative quotes for each theme in Table 2 and provided all other illustrative quotes in supplementary material.” raises different expectations: Table 2 I understand is now the framework without quotes. Table 3 (which I assume is meant) still has way too many quotes for them to be selective so that there is no substantial difference to the supplemental material. An exemplified selection of quotes would be capturable by the reader. In this context, quotes shall be transcribed under best practices (i.e. false starts and fillers should be deleted when overwhelming, i.e. “they have – how they have gone, how far have they gone”) – they may be left in the original version in the suppl. material but adapted in the illustrative quotes.
- I still believe that a road created is a very rare and not in all cases directly linked side effect of an educative project and hence inappropriate (in particular for the figure), also there are many more examples of infrastructure.
- The discussion encompasses some sentences which remain opaque to the reader even after several attempts (i.e. page 13 left column, upper part starting with “and to optimese this effect...” down to “...related to the single-level goal.”; or dto middle part “testing sites which were meaningful to those engaged in the GLP project”)
- Delete: page 13 left column, middle part “Future research...” until end of sentence
- Seems to be a repetition: page 13 left column middle part “Together, these findings...” until “...the research system itself.” and is taken up again in the last sentence of this column.
- The two numbers points (1. Use the three... and 2. Explicitly plan...) should be the end since they point to Box 1 (?) and could be part of the conclusion.
- The conclusion is yet another repetition. I trust the text could gain poignancy by revising both the discussion and conclusion.

**Typos and inconsistencies:**

- The language seems to become increasingly nonchalant towards the end of the text – please keep up high quality working throughout the text, i.e. the “GLP project” (see next point), increased redundancies, use of RCS in the discussion as acronym although never used nor spelled out before.
- Inconsistent use of the project name: whereas in the beginning there is no real mention of the project name, it seems to be referred to as “the GLP certification project” but then towards the end turns into slangish “’the GLP project’”.
- Page 3, second column, lower quarter: delete repetition of explanation of GLP which is already explained in the upper quarter of the column. Check for explanation of other acronyms
- Throughout the text and tables (i.e. table 1): correct and harmonize to “Côte d’Ivoire”, the CSRS is notoriously spelled out wrongly (change to read “scientifiques”), and I advise to check for correctness of names of all other organisations
- Consider revision of sentences: page 10 right column, middle, sentence starting with “This was suppported through...” seems to be incomplete;
- Inconsistencies in using “quality assurance” vs “Quality Assurance”,
- Further small typos (i.e. Table 2 effect to effects), please check for typos and don’t leave this to the reviewers
- Box 1: the header mentions “consideration” and three bullet points list them as considerations whereas most of the bullet points start with “consider” – reformulate to avoid considering considerations.

Is the work clearly and accurately presented and does it cite the current literature?

Yes

If applicable, is the statistical analysis and its interpretation appropriate?

Not applicable

Are all the source data underlying the results available to ensure full reproducibility?

Yes

Is the study design appropriate and is the work technically sound?

Yes

Are the conclusions drawn adequately supported by the results?

Yes

Are sufficient details of methods and analysis provided to allow replication by others?

Partly

Reviewer Expertise:

NA

I confirm that I have read this submission and believe that I have an appropriate level of expertise to confirm that it is of an acceptable scientific standard.

# Peer review: Reviewer response for version 1

**DOI:** 10.21956/gatesopenres.14395.r30103

**Published:** 2021-02-08

**Authors:** Elizabeth Hunsperger

**Affiliations:** 1 Centers for Disease Control and Prevention - Kenya Country Office, Nairobi, Kenya

The authors do not provide any quantitative data to support their findings. Although the authors fully describe the GLP process used to certify 5 laboratories in Africa continent spanning multiple countries, actual quantitative data to measure impact is lacking. The article is full of jargon and defining acronyms that is distracting from the main point. The article would benefit from editing and reducing the content to provide a concise report of the "ripple effect" of research in building lab capacity in these 5 countries. For example, table 1 is completely not helpful to the main point of the article. Similarly Table 2 is very long and overall not very informative. It seems to repeat most of the text. The most important point of this article is how the research activities improved on infrastructure of laboratories, this point gets lost in poor representation of these data or the lack of data.

 Overall this article would benefit significantly by trimming, removing tables and figures that are not informative and the addition of data to support the findings.

Is the work clearly and accurately presented and does it cite the current literature?

Partly

If applicable, is the statistical analysis and its interpretation appropriate?

No

Are all the source data underlying the results available to ensure full reproducibility?

No

Is the study design appropriate and is the work technically sound?

Partly

Are the conclusions drawn adequately supported by the results?

No

Are sufficient details of methods and analysis provided to allow replication by others?

No

Reviewer Expertise:

I am an arbovirologist working for CDC in global health in Kenya

I confirm that I have read this submission and believe that I have an appropriate level of expertise to state that I do not consider it to be of an acceptable scientific standard, for reasons outlined above.

# Response

**Published:** 2021-04-14

**Authors:** Sara Begg

**Affiliations:** Liverpool School of Tropical Medicine, UK

Thank you for taking the time to review this study. The study was designed to be qualitative as opposed to quantitative, and this is why there is no quantitative data presented here. The absence of quantitative data has been added to the limitations section. Comments on further amendments are available in our responses to the report provided by **Michael Käser**.

# Peer review: Reviewer response for version 1

**DOI:** 10.21956/gatesopenres.14395.r30104

**Published:** 2021-01-25

**Authors:** Adedayo O. Oduola

**Affiliations:** 1 Vector Biology and Control Research Group, Department of Zoology, University of Ilorin, Ilorin, Nigeria

The objectives of this study were clearly spelt out. This is a model that evaluates the contribution of capacity strengthening project on important stakeholders involved in the generation of quality and reliable scientific data that can influence good policy and decisions in vector control. The study provides useful information on  the effects and outcomes of the capacity strengthening on individuals and other relevant institutions. The study also contributes to existing knowledge by providing definite parameters that researchers can use to assess or monitor the impact of capacity building projects.

Is the work clearly and accurately presented and does it cite the current literature?

Yes

If applicable, is the statistical analysis and its interpretation appropriate?

Yes

Are all the source data underlying the results available to ensure full reproducibility?

No source data required

Is the study design appropriate and is the work technically sound?

Yes

Are the conclusions drawn adequately supported by the results?

Yes

Are sufficient details of methods and analysis provided to allow replication by others?

Yes

Reviewer Expertise:

Laboratory and field oriented vector control research. I am also into use of scientific  data to make sound public health decisions. I am also involved in capacity  strengthening of institutions involved in public health entomology research in Nigeria.

I confirm that I have read this submission and believe that I have an appropriate level of expertise to confirm that it is of an acceptable scientific standard.

# Response

**Published:** 2021-04-14

**Authors:** Sara Begg

**Affiliations:** Liverpool School of Tropical Medicine, UK

Thank you for taking the time to review this study, and we’re pleased that you have found this to be useful and relevant.

# Peer review: Reviewer response for version 1

**DOI:** 10.21956/gatesopenres.14395.r30105

**Published:** 2020-12-22

**Authors:** Michael Käser

**Affiliations:** 1 Swiss Tropical and Public Health Institute, Basel, Switzerland

This study analyses effects of a research capacity programme in non-clinical life science on various levels. For this, the authors compare five out of seven facilities in three African countries where GLP certification is being introduced to support vector control facilities that collaborate with each other. For this, they interviewed 65 staff, sampled by a maximum-variation purposive sampling strategy, in semi-structured interviews, for analysing effects on three levels: institutional, individual, and national/international. The results are being discussed in the context of the vector control centres and research capacity strenghtening (RCS) measures in general. The authors identify impact beyond the three levels which they describe as ripple effects.

 This report is another very nice example on assessing collaborative investment into research capacity, with a major resource allocation into people, and descriptively identifies details on those effects. Nicely, these effects are looked at on various levels, with a view on the interconnectivity between these layers. While they can be applied to similar research capacity building activities in the context of vector control, the transfer to other life science areas is being discussed. Surely, the community will learn from, and build upon, these experiences. Also, the study contextualizes the results into existing frameworks of RCS analysis not least through baseline consideration which renders the results more comparable.

 While it is not surprising that the study identifies positive side or unintended effects which are being named “ripple effects”, it is interesting to see how these additional effects are being carved out of the study participants’ views.

 This work should definitely be made available to the research community, in particular the one involved in (global) RCS, through publication in the proposed journal.

 However, the manuscript would benefit from revisions to gain clearness and improved readability, as described in the following:

**1. Methods**

- While 5 facilities were included into the survey, 7 facilities are involved and are being discussed; it is not always clear which number is referred to throughout the results and discussion.
- Since an interesting aspect of the work is the contextualization of the results to the existing frameworks, mention of it in the methods section would strengthen the methodology part.
- Some more information on the maximum-variation purposive/purposeful sampling should be added in addition to Ref. 19.
- Obviously the centres were investigated in their nature of facilities and equipment. Since it is assumed that these information do not stem from the survey, the description of the centres could be outlined in the methods.

**2. Content**

- The logic of the levels starts from institutional, presumably because this was the focus of the RCS activities under investigation. It then goes over individual to national/international level. This order is reflected in many of the passages and Table 1. In other sections, however, the order seems to follow the more natural one, starting with individual to continue with the institutional in the second place. This is seen in Table 2 and in Fig. 1-3 and in Box 1 and in some sections throughout the text (discussion page 14, second column). Harmonization could assist the reader in grasping the discussed points.
- The ripple effects: even when nicely carved out of the data, such effects are not so much of a surprise since they are identified in all RCS studies. The related content in the results is rather short and may explain the catchy word in the title but the discussion point seems to be a bit overstretched, i.e. through the description of transferrable skills. While unintended positive effects are expected in such RCS projects and were being described earlier, i.e. shared infrastructure such as roads may be caused by a mixture of reasons beyond RCS actions.
- Table 2 is hard to digest. It’s name “Target level for RCS” is unmentioned before, the abbreviation never used, “societal” used as synonym but never in the text. It appears that some 74 comments from the 66 survey participants were collected and grouped. While the entire table provides a wealth of opinions the minority of readers will be able to go through this table as such, so it could go into a supplementary table. The manuscript would benefit from the authors selecting categories and exemplifying representative citations to create a concise table with language-corrected statements by some survey representatives.
- Fig 1-3: While the message brought forward is clear, the figures create some unease when looking at: is a triangle the right geometric form for the levels as they imply either relative numbers or a basis on which other parts sit on. Even when the three messages are being understood, the reader identified somewhat redundant information: could they not combined into one (or a max of two), figures with the ripple effects being less prominently in design? Fig 3 implies much more data available. Fig 2: “institutional & national/individual relationship” is not clear and probably wrong.
- In addition to Ref 9, there should be mention of some more publications when bringing basic science facilities in context with RCS (Introduction). Also, when the point of ripple effects is being discussed (discussion), the point of unintended effects could benefit from citing additional evaluation studies of research capacity strengthening actions that Refs 22-26, i.e. https://pubmed.ncbi.nlm.nih.gov/27223888/ ^1^ or https://pubmed.ncbi.nlm.nih.gov/29216192/ ^2^ where clear ripple effects were shown even when not given that vocabulary.
- Throughout the text, there seem to be quite some redundancies, i.e. parts of the results (which could be also shortened) seem to be repeated in the discussion and parts of the discussion in the conclusions, and in particular when it comes to the ripple effects. Less repetition will not diminish the emphasis of this point.
- Limitations: as a limitation the lack of a quantitative analysis part to support the results should be mentioned.

**3. Minor points, typos, etc.:**

- key words: QM system and QM systems: redundant, depending on the journal’s search algorithm
- “The conducted of” consider revision (page 3, second column, line 5)
- PAMVERC-KCUMCo (page 4, acronym explained? even when one of the author’s affiliation)
- Côte d’Ivoire; National Institute For Medical...; (both page 4, same section of above comment)
- Research leaderhip/esteem – what is meant exactly? (page 14, second column)
- “suggests that further exploration of these effects is warranted” – what is meant exactly? (page 15, first column)
- Table 2: some are [Test Facility]s, others are [Collaborating Text Facility]s?; why is here the only mention of “societal” as obvious synonym of national/international?; order institutional/individual see comment above
- The community assumes that Consent Forms are also administered in French for the West African countries and in addition in local language, or reliably translated on site into the latter, as the version given is written in English – can this be reconfirmed by the authors?

Is the work clearly and accurately presented and does it cite the current literature?

Yes

If applicable, is the statistical analysis and its interpretation appropriate?

Not applicable

Are all the source data underlying the results available to ensure full reproducibility?

Yes

Is the study design appropriate and is the work technically sound?

Yes

Are the conclusions drawn adequately supported by the results?

Yes

Are sufficient details of methods and analysis provided to allow replication by others?

Partly

Reviewer Expertise:

Basic research, cell biology, immunology, molecular genetics Technology development and application, with focus on neglected tropical diseases (NTDs) Capacity building with focus on NTDs and in LMICs Evaluation of research capacity strengthening programmes

I confirm that I have read this submission and believe that I have an appropriate level of expertise to confirm that it is of an acceptable scientific standard, however I have significant reservations, as outlined above.

## References

[1] : Research Capacity Strengthening in Low and Middle Income Countries - An Evaluation of the WHO/TDR Career Development Fellowship Programme.*PLoS Negl Trop Dis*.10(5) : 10.1371/journal.pntd.0004631e000463110.1371/journal.pntd.000463127223888PMC4880212

[2] : Tracking the career development of scientists in low- and middle-income countries trained through TDR's research capacity strengthening programmes: Learning from monitoring and impact evaluation.*PLoS Negl Trop Dis*.2017;11(12) : 10.1371/journal.pntd.0006112e000611210.1371/journal.pntd.000611229216192PMC5736233

# Response

**Published:** 2021-04-14

**Authors:** Sara Begg

**Affiliations:** Liverpool School of Tropical Medicine, UK

Thank you for taking the time to review this study, we appreciate your input and experience in this area. We have made the following amendments to the manuscript based on your recommendations:

**Methods**

- Clarified throughout that effects described in this study are in the 5 test facilities involved in the study (described in Table 1) and highlighted where national/international level effects arose from the wider group of 7 test facilities.
- Added detail on the three-levels of research capacity strengthening used as a framework, in the methods section
- Added detail on the purpose and approach to sampling in this study (maximum-variation purposive/purposeful sampling)

**Content**

- Reordered methods, results and discussion so content always follows the order of individual, institutional and national/international level.
- Clarified that the extent to which ripple effects can be directly attributed to the RCS efforts is inevitably difficult since within a ‘real life research context there will inevitably be other factors that influence these effects. Stated that further exploration could be an area for future work.
- Selected two illustrative quotes for each theme in Table 2 and provided all other illustrative quotes in supplementary material. Two quotes were retained to ensure views of individuals in a diversity of different roles were still reflected within the main manuscript.
- Simplified figures into 2 figures; effects at the three levels and additional “ripple” effects, and illustration of the interrelated nature of the three levels in RCS efforts.
- Highlighted that unexpected/ripple effects have been previously observed in studies of individual level RCS efforts, and included additional references to support this.
- Simplified the text and removed repetition, particularly summaries of results within the discussion.
- Added acknowledgement of the absence of quantitate data in this study.

**Minor points:**

- Amended all of these, including additional confirmation on the language of consent documentation (available in French in West Africa, Swahili in Tanzania).
